# Logical and experimental modeling of cytokine and eicosanoid signaling in psoriatic keratinocytes

**DOI:** 10.1016/j.isci.2021.103451

**Published:** 2021-11-15

**Authors:** Eirini Tsirvouli, Felicity Ashcroft, Berit Johansen, Martin Kuiper

**Affiliations:** 1 Department of Biology, Norwegian University of Science and Technology, 7491 Trondheim, Norway

**Keywords:** Dermatology, Systems biology, Experimental models in systems biology

## Abstract

Psoriasis is a chronic skin disease, in which immune cells and keratinocytes keep each other in a state of inflammation. It is believed that phospholipase A_2_ (PLA_2_)-dependent eicosanoid release plays a key role in this. T-helper (Th) 1-derived cytokines are established activators of phospholipases in keratinocytes, whereas Th17-derived cytokines have largely unknown effects. Logical model simulations describing the function of cytokine and eicosanoid signaling networks combined with experimental data suggest that Th17 cytokines stimulate proinflammatory cytokine expression in psoriatic keratinocytes via activation of cPLA_2_α-Prostaglandin E_2_-EP4 signaling, which could be suppressed using the anti-psoriatic calcipotriol. cPLA_2_α inhibition and calcipotriol distinctly regulate expression of key psoriatic genes, possibly offering therapeutic advantage when applied together. Model simulations additionally suggest EP4 and protein kinase cAMP-activated catalytic subunit alpha as drug targets that may restore a normal phenotype. Our work illustrates how the study of complex diseases can benefit from an integrated systems approach.

## Highlights

• Th17 cytokines upregulate PGE_2_-EP4 signaling in psoriatic keratinocytes
• Inflammatory responses to Th17 cytokines can be suppressed using cPLA2α inhibitors
• Logical modeling is used to predict the effect of anti-psoriatic drugs
• Model analyses suggest EP4 and PRKACA as drug targets for treating psoriasis

## Graphical abstract

**Figure:**
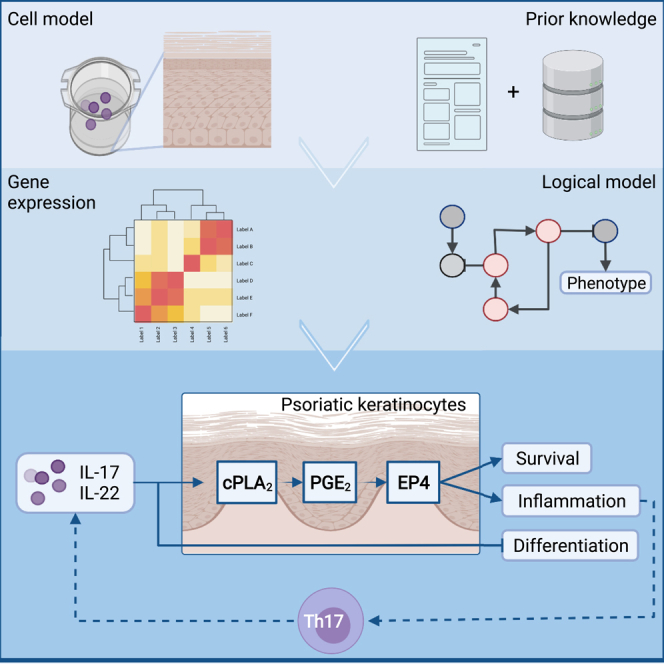

## Introduction

Psoriasis is a chronic inflammatory disease that affects 2%–3% of the world's population. Psoriasis vulgaris, commonly referred to as plaque-type psoriasis, is the most prominent type accounting for 90% of total cases (Rendon and Schäkel, 2019) and is associated with both environmental and genetic risk factors. Characteristic histological changes include thickening of the epidermis associated with hyperproliferation and aberrant differentiation of keratinocytes (KCs), an increase in dermal vascularity, and an infiltration of various immune cell types into the epidermis and dermis (Meephansan et al., 2017).

Psoriasis can be divided into two main stages: the initiation stage and the maintenance or chronic stage. Both stages are characterized by vicious positive feedback loops of signaling between keratinocytes and immune cells, where KCs respond to inflammatory triggers and act as a reservoir of pro-inflammatory mediators that promote immune cell infiltration (Benhadou et al., 2019). In the initiation phases, KCs respond to various environmental and genetic stimuli by secreting DNA-LL-37 or RNA-LL-37 complexes that recruit plasmacytoid DCs (pDCs) to the dermis and epidermis. The subsequent maturation of pDCs is marked by the production of high levels of interferon (IFN)-α. pDC-derived IFNα or RNA-LL-37 released by KCs activate myeloid DCs (mDCs), which in turn directly influence the differentiation of naive T cells (Albanesi et al., 2018; Benhadou et al., 2019; Rendon and Schäkel, 2019; Wang and Bai, 2020). In psoriasis, three T-helper (Th) cell subpopulations, namely Th1, Th17, and Th22 cells, have well-documented roles in the progression and maintenance of the disease (Casciano et al., 2018) by contributing to the cytokine milieu. Th1 cells release IFNγ and tumor necrosis factor alpha (TNFα), whereas Th17 cells produce interleukin-17 (IL-17) and IL-22, partially overlapping with the role of Th22 cells that produce IL-22 (Benham et al., 2013). Together, these cytokines stimulate hyperproliferation, premature differentiation, and resistance to apoptosis in KCs, largely through the activation of signal transducer and activator of transcription 3 (STAT3) and nuclear factor kappa B (NF-κB) transcription factors (Albanesi et al., 2018). Their effects are, however, not limited to changing keratinocyte physiology but also include the stimulation of KCs to produce antimicrobial peptides (AMPs), proinflammatory cytokines, and chemoattractants, which together results in neutrophil recruitment and the maintenance of the same Th-cell populations. Thus, KCs participate in a positive feedback loop with the immune system, which sustains chronic inflammation (Albanesi et al., 2018; Deng et al., 2016; Georgescu et al., 2019).

Psoriasis is currently incurable, and therefore long-term treatments able to cope with the relapsing episodes of the disease are important (Gisondi et al., 2017). Advancements in our understanding of the disease have led to the development of biologics targeting, for example, IL-17 and TNFα (Bellinato et al., 2021), which are used systemically to treat moderate and severe disease. Topical treatment options for milder disease include calcipotriol, which is given either alone or in combination with the corticosteroid betamethasone dipropionate (Fenton and Plosker, 2004). Calcipotriol is an analogue of vitamin D, and its therapeutic benefits are thought to occur via effects on both KCs and immune cells (Kim, 2010). Current treatment options appear to have relatively high efficacy and tolerability; however, immediate or long-lasting efficacy can vary between patients (Kamata and Tada, 2020; Kim, 2010). In addition, certain biologics are accompanied by side effects and tolerability issues such as recurrent infections (Kamata and Tada, 2020). Combination treatment with two or more agents has been proposed as a way to overcome these challenges and improve efficacy in nonresponding patients, while ameliorating and reducing the potential side effects of long-term treatments (Cather and Crowley, 2014).

Type A_2_ phospholipases (PLA_2_) are a diverse family of enzymes that catalyze the hydrolysis of membrane phospholipids producing fatty acids and lysophospholipids. They are typically categorized into six types: cytosolic (cPLA_2_), calcium independent (iPLA_2_), secreted (sPLA_2_), lysosomal (LPLA_2_), adipose (aiPLA_2_), and the platelet-activating factor acetyl-hydrolases (PAF-AH) with highly varied physiological roles. Dysregulation of PLA_2_ activity has been demonstrated in multiple diseases, including cancer, rheumatoid arthritis, and psoriasis (Leistad et al., 2011; Linkous and Yazlovitskaya, 2010; Magrioti and Kokotos, 2013), and drugs targeting PLA_2_s are being considered for treating mostly inflammatory diseases (Nikolaou and Kokotos, 2018). One form, cPLA_2_ɑ has attracted particular attention as a therapeutic target for the treatment of both hyperproliferative and inflammatory diseases (Yarla et al., 2016). This is due to its key role in the generation of bioactive lipid species and fatty acids, including arachidonic acid (AA) and lysophospholipids in response to growth factors and pro-inflammatory cytokines, dependent on intracellular calcium release and protein phosphorylation (Niknami et al., 2009). Upon activation, it specifically hydrolyzes phosphatidylcholine (PC) to yield AA and lysophosphatidylcholine (LPC). AA can be further metabolized by cyclooxygenase (COX) and lipoxygenase (LOX) enzymes to produce prostaglandins (PGs), leukotrienes (LTs), and hydroxy fatty acids, e.g. 12s-HETE, whereas LPC can be further metabolized to platelet-activating factor (PAF). Both AA metabolites, or eicosanoids, and PAF play important roles in diverse physiological processes including immune responses, pain, and cell growth but also contribute to the pathogenesis of various diseases, often having pro-inflammatory effects, but also via the activation of survival-promoting kinases (Hirabayashi et al., 2004; Linkous and Yazlovitskaya, 2010; Murakami et al., 2017).

Epidermal KCs are an important source of eicosanoids (Nicolaou, 2013) and PAF (Pei et al., 1998), which can participate in the infiltration and amplification of immune cells (Camp et al., 1984; Lee et al., 2019; Sheibanie et al., 2004; Ueharaguchi et al., 2018) in addition to acting upon the KCs themselves via G-protein-coupled prostanoid, leukotriene, and PAF receptors (Kanda and Watanabe, 2007; Kim, 2010; Konger et al., 1998, 2005; Southall et al., 2001). Several studies show that prostaglandin E_2_ (PGE_2_) can shift adaptive immunity toward Th17 and Th1 responses by affecting DCs (Chizzolini and Brembilla, 2009), regulating the priming and expansion of Th17 cells, and promoting the differentiation of Th1 cells (Boniface et al., 2009; Napolitani et al., 2009; Yao et al., 2009). PGE_2_ acts through four receptors, EP1–EP4, all expressed in KCs (Rundhaug et al., 2011). EP4, which activates both the AC/cAMP pathway and the PI3K/AKT pathway (Rundhaug et al., 2011), and EP2 are shown to mediate the observed responses to PGE2 relevant to psoriasis (Tsuge et al., 2019). The roles of EP1 and EP3 in psoriasis have not been well studied; however, their associated phenotypes do not appear to be characteristic for the disease, as EP1 was found to promote keratinocyte differentiation in nonmelanoma skin cancer (Konger et al., 2009), and EP3 was proposed as a growth inhibitor for KCs (Konger et al., 2005). Thus, inhibiting cPLA_2_α presents a promising target for treating psoriasis via suppressing the production of PGE_2_ and potentially other bioactive lipids, and this is supported by the promising effects of a topical cPLA_2_α inhibitor, AVX001, in clinical trials against mild to moderate psoriasis (Omland et al., 2017).

Addressing the limited understanding of the role played by cPLA_2_ɑ and eicosanoid signaling in the activation of psoriatic keratinocytes and the development of the disease would benefit from a unified framework that collects prior knowledge and integrates new observations to produce a modeling tool that can be further used to not only make sense of observations but also rationalize experimental decisions, test hypotheses, and highlight gaps in our knowledge. In silico approaches to predict and interpret the effects of stimuli and perturbations in cellular systems have become increasingly relevant. Among different possible approaches, logical models that represent the signaling events and causal interactions between cellular components prove to be able to capture the behavior of cells in experiments, predict the effect of drugs, and identify potentially synergistic combinations (Flobak et al., 2015; Pirkl et al., 2016; Tsirvouli et al., 2020). In this study, we aimed to develop such a logical model to represent the complex signaling events occurring in psoriatic keratinocytes to (1) investigate the mode of action of a cPLA_2_ɑ inhibitor, (2) predict therapeutic benefits of drug combinations, and (3) investigate the contributions of Th17 versus Th1 cytokines to disease phenotypes.

## Results

We set out to develop an in silico model of psoriatic KC that could be used to describe the development of cytokine-dependent phenotypes of psoriatic KCs and the therapeutic mechanism of action of cPLA_2_α inhibitors alone and in combination with other anti-inflammatory agents. The construction of the logical model started with describing a range of experimental observations regarding the eicosanoid signaling taking place in Th17-cytokine-stimulated KCs that the model should reconcile. Prior knowledge together with these experimental observations was integrated into a regulatory network, which was then used to simulate the response of KCs to external stimuli and predict their effect on the psoriatic environment with respect to the secretion of ligands and intercellular-acting stimuli.

### Th17 cytokines regulate KC differentiation and prostaglandin E2 release *in vitro*

*In vitro* models of psoriasis can be created by exposing monolayers or 3D skin-equivalent cultures to psoriatic cytokines (Desmet et al., 2017). We have previously used the immortalized KC cell line, HaCaT, in 3D skin-equivalent cultures to document the role of cPLA_2_α in KC proliferation (Ashcroft et al., 2020). These cells express both cPLA_2_α and sPLA_2_ and have been used to study the regulation of eicosanoid production by Th1 cytokines in the skin (Sjursen et al., 2000; Thommesen et al., 1998). HaCaT cells are also known to respond to Th17 cytokines (Woo et al., 2017) but the role of cPLA_2_α in mediating the effects of Th17 cytokines in KCs is not known.

**Figure 1:**
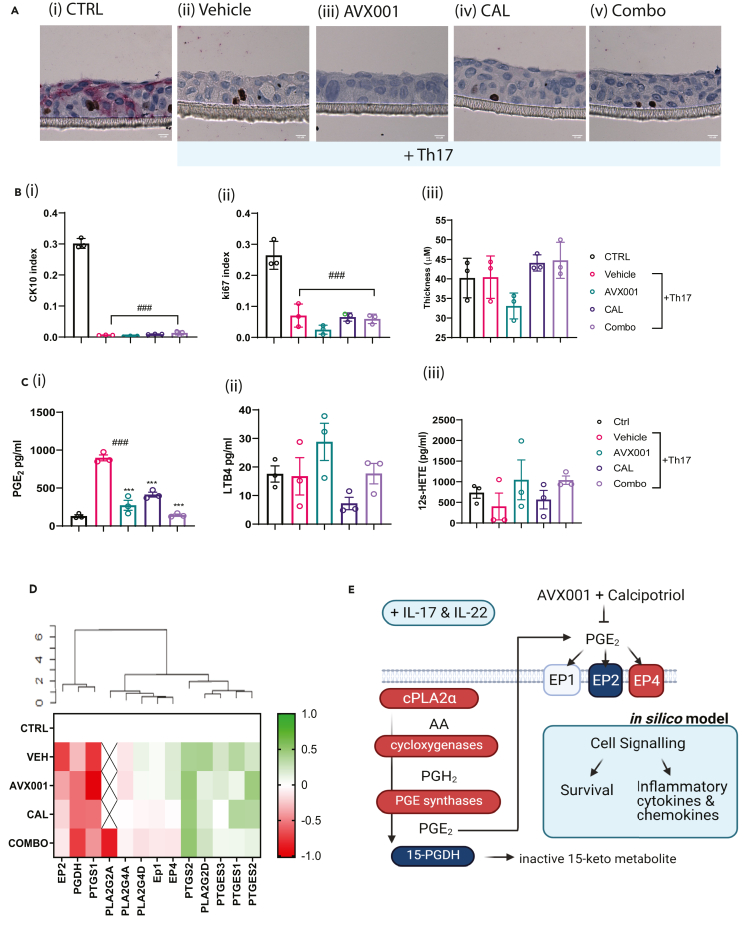
Regulation of cPLA_2_/PGE/EP4 signaling by Th17 cytokines (A) Representative images showing immunostaining with anti-Ki-67 (DAB+) and anti-cytokeratin 10 (Permanent Red) antibodies. Scale bar, 10 μM. (B) Quantification of (i) the proportion of CK10 positive cells, (ii) the proportion of proliferating cells, and (iii) the thickness of the cultures. The data shown are the mean ± SEM for three replicates. One-way ANOVA followed by Dunnett's multiple comparison test was used to make statistical comparisons with either the untreated control (CTRL) or stimulated control (Vehicle), where ### indicates p < 0.001 versus CTRL (C) Eicosanoid levels measured by ELISA for (i) PGE_2_, (ii) 12s-HETE, and (iii) LTB_4_. Data shown are the mean ± SEM of three replicates, measured in duplicate. One-way ANOVA followed by Dunnett's multiple comparison test was used to compare the PGE_2_ levels with either the untreated control (CTRL) or the stimulated control (Vehicle), where ### indicates p < 0.001 versus CTRL and ∗∗∗ indicates p < 0.001 versus Vehicle. (D) Heatmap of Log_10_ fold changes in expression relative to unstimulated controls (CTRL) for genes involved in PGE_2_ synthesis, degradation, and signaling. Data are the mean of three replicates. X indicates that expression was undetectable. (E) Schematic summary showing how Th17 cytokines could regulate PGE_2_ release and activate downstream signaling events via the EP4 receptor. The gene expression of the components in red was increased and decreased for components in blue in cultures treated with Th17 cytokines.

To investigate the role of cPLA_2_α in Th17-dependent signaling in KCs we used air-exposed 3D cultures of HaCaT grown in the presence of a combination of IL-17 and IL-22 (from here on referred to as Th17 cytokines). Immunohistochemical staining of fixed, paraffin-embedded cultures was used to quantify proliferating and differentiating cells with Ki67 and cytokeratin 10 (CK10) positivity, respectively. Eicosanoid release was measured by ELISA. The control cultures were 140–180 μm thick, typically comprising 4–5 cell layers. Approximately 20% of the cells were Ki67 positive, consistent with being in a proliferative state, whereas approximately 30% were CK10 positive, indicating early differentiation. Treatment with Th17 cytokines did not affect the thickness of these cultures but, consistent with several studies (Boniface et al., 2009; Nograles et al., 2008; Pfaff et al., 2017; Rabeony et al., 2014), affected differentiation, as evidenced by the loss of CK10 expression. The Th17-treated cultures also had significantly reduced Ki67 positivity, indicating lower proliferation, which is inconsistent with the hyperproliferative state of the psoriatic epidermis. Treatment with either AVX001, CAL, or COMBO had no effect on the thickness, CK10, or Ki67 positivity of the Th17-treated cultures (Figures 1A and 1B).

Increased levels of the prostaglandin PGE_2_ were measured in supernatants from cultures treated with Th17 cytokines, whereas leukotriene B4 (LTB4) and 12s-HETE were not affected. Suppression of cPLA_2_α activity using the cPLA_2_α inhibitor AVX001 (Huwiler et al., 2012) reduced the PGE_2_ levels implicating the activation of the enzyme in this response. Similarly, the vitamin D analogue and topical antipsoriatic drug calcipotriol (10 nM) reduced the PGE_2_ levels, and when we combined the two compounds (COMBO), PGE_2_ levels were similar to the unstimulated controls (Figure 1C).

### Th17 cytokines regulate PGE2 biosynthesis and signaling in KCs *in vitro*

The biosynthesis of PGE_2_ results from the coordinated regulation of several enzymes. Free arachidonic acid (AA) released as a result of phospholipase A_2_ activity is metabolized to PGH_2_ by the activity of cyclooxygenases, Cox-1 (*PTGS1*) and Cox-2 (*PTGS2*), which is further metabolized to PGE_2_ by prostaglandin synthases (*PTGES1, PTGES2, PTGES3*). Intracellular PGE_2_ can be oxidized to an inactive 15-keto form by the enzyme 15-prostaglandin dehydrogenase (PGDH) or released into the extracellular space where it is an autocrine or paracrine ligand for the eicosanoid-prostaglandin (EP) family of receptors (*PTGER1-4)* residing at the cell surface. Although psoriatic lesional skin has been shown to overexpress enzymes involved in PGE_2_ biosynthesis and underexpress *15-PGDH* (Lee et al., 2019), the separation between regulation of the PGE_2_ biosynthesis pathway in KCs versus infiltrating immune cells was not made.

To investigate how Th17 cytokines regulate of PGE_2_ production and signaling for integration into the computational model, we measured the expression of several genes involved in the biosynthesis and downstream recognition of PGE_2_. Total RNA was extracted from the HaCaT cultures treated with Th17, in the absence or presence of AVX001, calcipotriol, or a combination of AVX001 and calcipotriol (COMBO). The expression of *PLA2G4A*, *PTGS1*, *PTGS2*, *PTGES1-3*, *15-PGDH*, four prostaglandin E receptors *(PTGER1-4)*, and three internal reference genes (*TBP*, *HPRT1*, and *GAPDH*) were measured by quantitative polymerase chain reaction (qPCR)*.* Results are shown relative to the unstimulated control (CTRL) (Figures 1D and S1). Hierarchical clustering identified three groups characterized by upregulation, downregulation, or a lack of regulation by Th17 cytokines. *PTGS2* and *PTGER4* abundances were increased, whereas *PTGS1*, *PTGER2*, and *15-PGDH* expression levels were reduced. A trend toward the increased expression of prostaglandin E synthases was also observed, although no changes reached statistical significance at p < 0.05. With the possible exception of *PTGES3*, calcipotriol treatment did not reverse the effects of IL-17 on *PTGS*, *PTGES*, or *15-PGDH* expression, indicating suppression of PGE_2_ release by calcipotriol may occur via an independent mechanism. Interestingly, both calcipotriol and COMBO treatments rescued the effect of Th17 cytokines on *PTGER4* and *PTGER2* expression.

In summary, Th17 cytokines stimulated PGE_2_ release from KCs, which was dependent upon cPLA_2_α activity and was associated with increased expression of *PTGS2* and decreased expression of *15-PGDH*. Th17 additionally suppressed *PTGER2* and increased *PTGER4* expression. Combining AVX001 with calcipotriol completely suppressed Th17-dependent PGE_2_ release and reversed the switch from *PTGER2* to *PTGER4* expression (summarized in Figure 1E). These findings were integrated into the in silico keratinocyte model as Th17 cytokine-dependent activation of cPLA_2_ɑ/COX-2/EP4 signaling as described below.

### The logical model of psoriatic KCs

**Figure 2:**
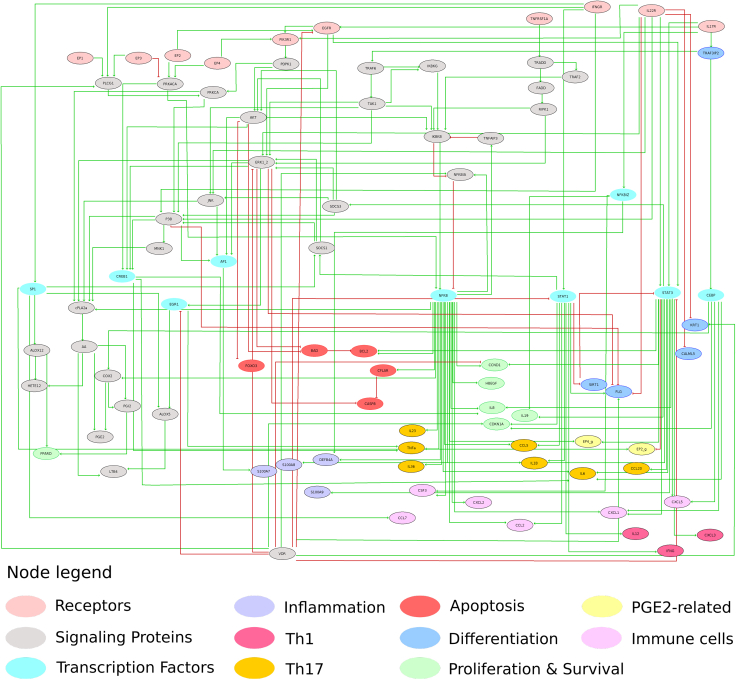
The logical model of psoriatic keratinocytes The psoKC model represents (i) the cell fate decisions of keratinocytes (i.e. proliferation, apoptosis, differentiation) in response to IFNγ, TNFα (mainly produced by Th1 cells), IL-17, IL-22 (mainly produced by Th17 cells), and PGE_2_ and (ii) the influence of keratinocytes on the psoriatic environment by characterizing the secreted ligands (e.g. chemokines and cytokines), which would be produced by KCs and influence immune cells by sustaining their populations and inflammation. The node color depicts their functional role, the phenotype they promote, or the immune cell types they act on. Green lines represent activating interactions, and red lines represent inhibitory interactions.

To recapitulate all these observations in a consistent regulatory model that captures as much as possible the deregulatory events leading to psoriasis, we focused on the representation of the KCs as responders to Th-cell-derived cytokines. The psoriatic keratinocyte (psoKC) model is presented in Figure 2. The model aims to integrate the available knowledge on regulatory interactions that take place during the chronic stages of psoriasis, including the newly described regulation of PGE_2_ signaling. The model contains 88 biological entities (nodes) and 170 regulatory interactions (edges) and can be stimulated by the activation of the receptors recognizing the main psoriatic cytokines, namely IL-17, IL-22, TNFα, and IFNγ, and PGE_2_ (EP) receptors. It is important to highlight that the regulatory mechanism of cPLA_2_ɑ in the system is mainly designed to be able to assess the effect of PGE_2_ through the EP receptors. This effect is encoded in a way that the activation of EP receptors is an input and not directly activated by the PGE2 node in the model. To account for the autocrine effects of PGE_2_ in KCs, the EP receptors were set to be active in the analyses where cPLA_2_ɑ activity is uninhibited. To distinguish the EP receptor nodes from their genes, which are activated by different transcription factors downstream in the model, a suffix *_g* was added to the nodes that represent their respective genes. The description of the model nodes and their logical rules can be found in Table S2 in the supplemental information. The model was deposited in BioModels (Malik-Sheriff et al., 2020) and assigned the identifier BioModels: MODEL2109300001.

The model can describe the three dysregulated phenotypes of KC in psoriasis: hyperproliferation, resistance to apoptosis, and aberrant differentiation. The model furthermore covers the KC's immunostimulatory states, as manifested by the production of cytokines, chemokines, and AMPs (antimicrobial peptides) that activate, recruit, and maintain immune cell populations that contribute to the psoriatic phenotype. The aforementioned physiological states that the model is able to represent under different simulated conditions can be inferred from the state (ON or OFF) of selected sets of markers that are characteristic of each specific cellular phenotype. The marker nodes were defined and selected as those entities that are included in the transcriptional signatures of the psoriatic cytokines on KCs and associated with one or more phenotypes that the model aimed to represent. The node-cellular state association can be seen in Table S3 in the supplemental information.

### Integration of experimental observations into the model and validation with *in vitro* results

Our experimental observations suggest that cPLA_2_ɑ/PGE_2_/EP4 signaling is active in response to Th17 cytokines in KCs. The potential involvement of this pathway in the development of psoriasis was tested in stable state calculations by comparing the states of phenotypic marker nodes when EP4 was active and inactive. In conditions where EP4 was inactive, CFLAR, CREB1, IL-8, CSF3, DEFB4A, IL-23, and IL-36 genes were also predicted to be inactive. However, when EP4 was encoded to be active, the states of the aforementioned entities were corroborating the available literature on the state of these entities in psoriasis.

**Figure 3:**
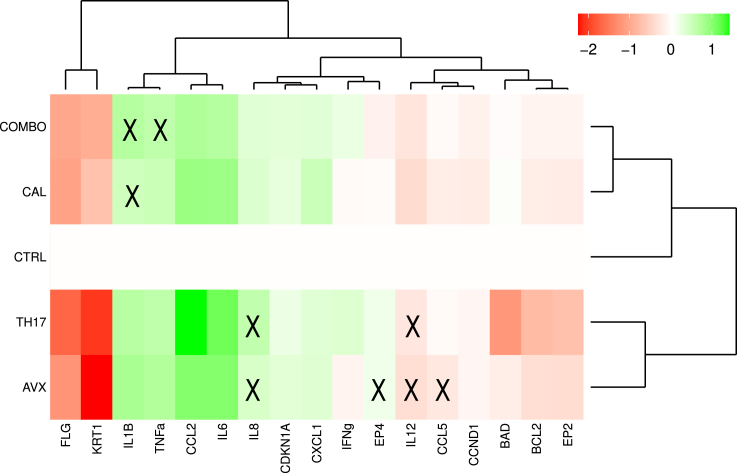
Gene expression levels of selected marker nodes and comparison with node states predicted by the computational model Hierarchically clustered heatmap of log_10_ fold change in gene expression relative to CTRL as determined by qPCR. HaCaT 3D cultures were treated with Th17 cytokines (TH17) in the presence of AVX001 (AVX), calcipotriol (CAL), or a combination of AVX001 and calcipotriol (COMBO). “**X**” represents markers for which the model predicted a different state than their experimentally observed activity.

In order to further investigate the role of cPLA_2_ɑ/PGE_2_/EP4 signaling experimentally, the expression of 17 phenotypic marker genes (Shown in Table S3) was measured by qPCR. Th17 cytokines increased the gene expression levels of pro-inflammatory cytokines (*IL6*, *IL1β*, *TNF-α*, *IL-8*, *CCL2*, *CXCL2*) and decreased the expression genes associated with differentiation (*FLG*, *KRT1*) and apoptosis (*BAD*) (Figures 3 and S2). Inhibition of the cPLA_2_ɑ using AVX001 or treatment with calcipotriol alone suppressed the induction of *CCL2* and prevented the inhibition of *BAD*. Calcipotriol additionally caused a partial rescue of the loss of *KRT1* expression. Combining AVX001 with calcipotriol inhibited more of the proinflammatory markers (*CCL2*, *IL-6*, and *IL-8*). To compare these experimental results to the Boolean states of the corresponding nodes in the model, the gene expression data were discretized to “ones” and “zeros” (Described in Figure S3). For 59 of the 68 experimental observations, the model predictions were in agreement (Figure 3). Interestingly, the nine observed discrepancies were seen only in perturbed simulations, with four of these nine discrepancies occurring with the use of the cPLA_2_ɑ inhibitor alone.

### Predicting keratinocyte behavior upon different stimuli and treatments

Encouraged by the apparent value that the logical model has in predicting verifiable experimental results, we performed a series of stable state calculations in an effort to gain further insights into the regulatory mechanisms underlying psoriasis. Although it has been suggested that different Th cells are dominating and controlling the inflammatory process during the various stages of the disease (Furiati et al., 2019), the notion that Th1, Th17, and Th22 cells contribute to the development of psoriasis has become increasingly accepted (Diani et al., 2016). The downstream analyses and experimentations were focused on gaining a better understanding of how different sets of cytokines, and by extension different cell types, affect the behavior of KCs, how this behavior changes when all cytokines are present, and, lastly, how PGE_2_-regulated signaling is integrated into the system.

**Figure 4:**
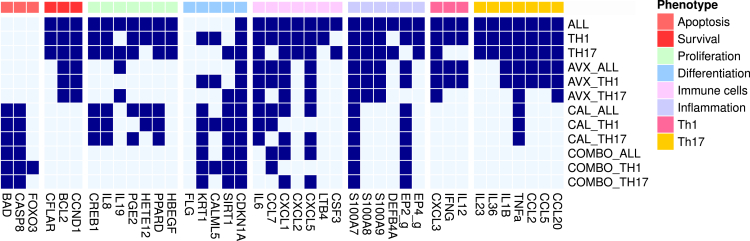
Heatmap of system's perturbations The heatmap depicts the set of active and inactive marker nodes, grouped by their associated phenotype, in each simulated condition. Light blue denotes inactive entities, whereas dark blue denotes active entities, the number which can be taken as a measure of compliance with specific phenotypes.

The response of KCs to stimuli from Th1 (i.e. IFNγ and TNFα), Th17 (i.e. IL-17 and IL-22), and Th1 and Th17 combined was simulated. For the same conditions, the effect of chemical perturbations with cPLA_2_ɑ inhibitors, vitamin D analogues, or a combination of the two was simulated. Similar to experimental assays, as for example in apoptotic assays where the activation of Caspases is measured as an indication of apoptosis, the phenotypes that the model reached under different simulated conditions were inferred by the state of phenotype-specific markers. For that reason, the simulation results are presented as a series of active and inactive markers for each condition in Figure 4. All perturbed conditions (i.e. AVX, CAL, and COMBO) were able to reach a single stable state, a phenomenon that can be attributed to the absence of positive regulatory circuits (Thieffry, 2007), as identified by a functional circuit analysis performed on the logical model.

As displayed in the first three rows of Figure 4, which represent unperturbed conditions, the model predicts that all cytokines contribute to the maintenance and amplification of the positive feedback loop between KCs and Th cells. However, it is only when all four cytokines are present (ALL condition in Figure 4) that all immunomodulatory markers are activated. The substantial overlap between the Th1 and Th17 conditions could indicate that the two sets of inputs act synergistically rather than complementarily, as similar observations were made when analyzing the synergistic effect of IL-17, TNFα, and IFNγ (Chiricozzi et al., 2011). Interestingly, the inhibition of cPLA_2_ɑ (rows 4–6) mainly affects proliferation markers and only some inflammatory and immune cell markers. These results agree with previous studies of the role of PLA2 enzymes and their downstream products in psoriasis (Ashcroft et al., 2020) and skin biology in general (Murakami et al., 2017). Moreover, it is worth noting that the targeting of cPLA_2_ɑ appears to mainly act on the Th17-cell-related markers, but not on the Th1 markers. However, as the model is mainly focusing on the role of PGE_2_ in the system, it is possible that other cPLA_2_ɑ downstream products affect Th1 cell markers but that their associated mechanisms are not depicted in the current version of the model.

When comparing the effect of cPLA_2_ɑ inhibition with the effect of calcipotriol (rows 4–6 and 7–9 in Figure 4), it is evident that both drugs have a distinct mechanism of action, as calcipotriol appears to affect a different set of markers than cPLA_2_ɑ. More specifically, calcipotriol affects mostly differentiation and apoptosis, whereas cPLA_2_ɑ appears to mostly affect proliferation. Calcipotriol is able to have both an antiproliferative (Kristl et al., 2008; Liang et al., 2017) and a pro-apoptotic effect (Huang et al., 2019; Tiberio et al., 2009) in psoriatic KCs. Apoptosis is subject to an elaborate regulation and controlled by the ratio of pro- to anti-apoptotic regulators, meaning that both types of regulators can be active at the same time (Jan and Chaudhry, 2019). As seen in Figure 4, calcipotriol appears to shift the balance toward pro-apoptotic regulators and appears to promote apoptosis in the system, regardless of the input conditions. At the same time, proliferation-promoting eicosanoids, such as PGE_2_ and 12s-HETE, are predicted to still be produced, unless cPLA_2_ɑ is inhibited. The same behavior has been reported in various dying cells, where PGE_2_ is released as a damage-associated molecular pattern (Hangai et al., 2016). Keratinocyte differentiation is also rescued by calcipotriol, as indicated by the states of the differentiation markers. Interestingly, terminal differentiation of KCs shares many similarities with apoptotic mechanisms (Terskikh and Vasil'ev, 2005), as it can also be seen in the coordinated change of both sets of markers.

Finally, as seen in the COMBO stable states, a distinct change of the markers' behavior is evident, both in the KC cell fate and the suggested effect of the KC cell on immune cell behavior. Although we cannot directly quantify the effect of the different drugs and their combination, it is clear that the combination of the two drugs results in what appear to be additive changes in the system. cPLA_2_ɑ inhibitor and calcipotriol together inhibit all endogenous and exogenous markers that would promote proliferation and survival. Furthermore, the recruitment of immune cells appears substantially impaired (absence of chemoattractants and immunostimulatory cytokines), whereas primary proinflammatory cytokine TNFα is also inhibited when both drugs are used. The effect on differentiation, however, can be attributed solely to calcipotriol, as the additional cPLA_2_ɑ inhibition in the combo treatment has no effect on these phenotype markers.

### Evolution of the regulatory system through “time” and phenotype probabilities

A stable state analysis, as the one presented earlier, provides great insights into which stable states, intuitively analogous with cellular phenotypes, a system can occupy. In stable states, the model components “stabilize” in either an active or an inactive state. In order to approximate the observation of transient activation or inactivation of nodes, and the general evolution of the nodes' states until the model reaches a stable state, stochastic simulations were performed. Such analysis results in the probabilities of the model reaching a certain stable state/phenotype, as well as how the node states evolve toward a final stable state.

**Figure 5:**
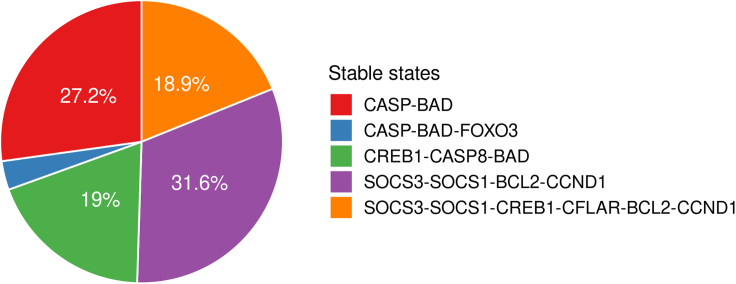
Stable state probability pie chart Probabilities of stable states after the stimulation with IFNγ and TNFα in an unsynchronized population of cells as calculated by stochastic simulations. SOCS1 and SOCS3 were identified as the markers whose activation is associated with resistance to cytokine-mediated apoptosis.

As observed in our *in vitro* experiments and reported in the literature (Boniface et al., 2005; Ekman et al., 2019; Pfaff et al., 2017), stimulation by Th17 cytokines, and mainly IL-22, completely suppresses differentiation and apoptosis in KCs, with survival and anti-differentiation markers being active and differentiation markers inactive with a 99.9% probability in the stochastic simulations. The promotion of an anti-apoptotic phenotype by Th1 cytokines was also confirmed by stochastic simulations, where an anti-apoptotic phenotype was reached with 100% probability (Figure S4). Nevertheless, other studies have shown that under certain conditions, TNFα and IFNγ can induce apoptosis in KCs (Reinartz et al., 1996; Viard-Leveugle et al., 2013). To see if this behavior was dependent on the state of specific components of the system, simulations with active TNFα and IFNγ were performed with an exhaustive series of initial conditions where all entities had a 50% chance of being active. Although the system was now able to reach several states with different sets of markers being active each time, the states could be separated into apoptosis and survival. By analyzing the difference between those states, two proteins seemed to be determinants for whether apoptosis would be reached: SOCS1 and SOCS3. Cell populations with active SOCS1 and SOCS3 were able to escape apoptosis (Figure 5). Both proteins were found overexpressed in psoriatic skin, compared with normal skin (Federici et al., 2002), and their activity may be contributing to the resistance of psoriatic KCs to cytokine-induced apoptosis, via a mechanism involving the activation of PI3K/AKT and NF-κB pathways (Madonna et al., 2012). Simulations of the dynamic interplay and temporal evolution of the states pro-apoptotic and anti-apoptotic markers revealed that the combinatorial treatment, which inhibits the survival markers, renders pro-apoptotic marker activity unconstrained so that they reach a final active stable state (Figure S5). Lastly, in stochastic simulations where all nodes had equal probabilities of being active or inactive, a direct correlation between the probability of activation of CASP8 (i.e. Caspase-8) and the probability of reaching an apoptotic phenotype was observed. This observation could be described as a logical consequence of the dependence of apoptotic phenotype in the irreversible activation of the caspases. Indeed, in a real-life scenario, a cell with an already activated caspase would be expected to undergo apoptosis irrespectively of the additional stimuli.

### Understanding the impact of each cytokine in the system

To explore in more depth the mechanistic regulation of different inputs that leads to the distinct phenotypes identified by the previous analysis, a “value propagation” analysis was performed, allowing us to trace in detail the effects of various inputs through the regulatory graph. An analysis of these effects can be used to identify if the activation of certain inputs (i.e. stimuli) is enough to set the state of a node as active or inactive, without the need of additional stimuli. A comparison of the effects of the inputs should then indicate whether one or more cytokines that derive from the same Th-cell population are sufficient to induce characteristic psoriatic phenotypes or whether it is rather the integration of the stimuli from different Th-cell populations that results in these phenotypes.

**Figure 6:**
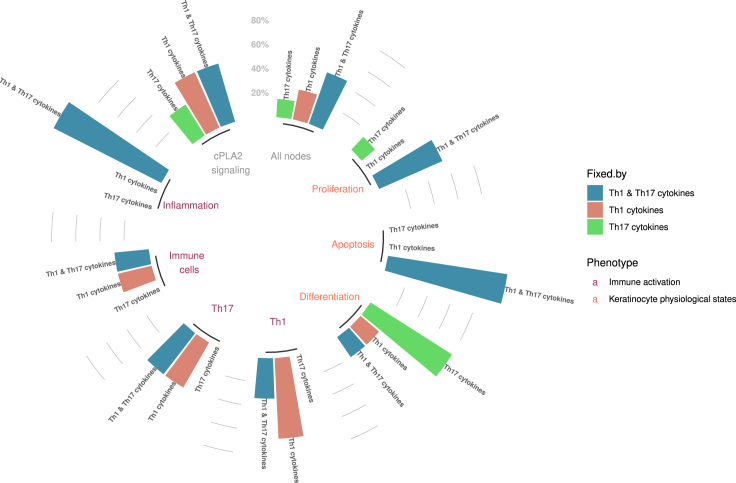
Impact of cytokines in keratinocyte phenotypes and physiological states as identified by a value propagation analysis The height of each bar denotes the percentage of the fixed nodes out of the total marker nodes for each phenotype. Empty bar slots correspond to 0% fixed nodes by a certain stimulus. All nodes refer to both markers and intermediate signaling nodes.

The analysis revealed that stimulation with the Th1-derived cytokines impacted more phenotypic markers than stimulation with IL-17 and IL-22 (see “All nodes” in Figures 6 and S6), as also seen in the stable state analysis. This result agrees with transcriptomic studies that have shown a dominance of Th1 and, more specifically, of IFNγ signature expression profiles in psoriatic lesions (Albanesi et al., 2018). However, the transcription of genes of key inflammatory mediators, such as S100 antimicrobial peptides, IL-6, and TNFα, which act synergistically to maintain an inflammatory response in psoriasis, can be activated by both sets of cytokines. The effects of the combination of IL-17 and IL-22 on differentiation and proliferation in the system that was observed in the stable state analysis (see Figure 4) and *in vitro* experiments (see Figure 1) are confirmed by the value propagation study, where their simulation primarily fixes the activities of differentiation and proliferation markers to states that are associated with a repressed differentiation phenotype. Conversely, IFNγ and TNFα mainly fix the state of a group of nodes that is associated with Th cell maintenance. At the same time, all cytokines together lock inflammatory nodes in their active state.

As cPLA_2_ɑ is of special interest as a psoriatic drug target, cytokine effects on eicosanoid production were analyzed in more detail. Although all cytokines appear to be able to activate the phospholipase, the inducible enzyme COX-2, which catalyzes the production of PGE_2_, is activated only downstream of IL-17 and IL-22, corroborating our *in vitro* observations. Conversely, TNFα and IFNγ induced the expression of LTB4 and 12-HETE by regulating their respective enzymes. Both LTB_4_ and 12-HETE are reported to play a role in the pathogenesis and development of the disease and have chemotactic properties (Nicolaou, 2013), again confirming the immunostimulatory action of Th1 cytokines.

A comparison of the distinct effects of IL-17 and IL-22 revealed a greater overall impact of IL-17 on the regulation of both signaling proteins and markers (Figure S7), an observation that also finds support in the literature (Nograles et al., 2008; Rabeony et al., 2014). Nograles et al. also reported that IL-22, but not IL-17, regulated terminal differentiation markers of KCs (i.e. CALML5, KRT1, and FLG). Indeed, upon activation of IL-22, CALML5 and KRT1 are fixed in an inactive state, whereas proliferation and anti-differentiation markers (i.e IL-6 and IL-29) are active. However, a clear distinction of their role was not observed in our model analysis because both cytokines appear to have an impact on the immunostimulatory markers. In the comparative value propagation between Th1 cytokines (Figure S8), it was found that although IFNγ dominates the regulation of immunostimulatory markers, TNFα has a more limited impact.

### Model-based analyses to assess possibilities for the treatment of psoriasis

The availability of a logical model representation of psoriatic KCs allows a model analysis that supports an exploration of the possible perturbation space to search for model nodes that may serve as potential drug targets in treatments that could restore a normal phenotype. A full analysis of the perturbation space was performed, with each entity being considered a potential drug target, to allow the identification of novel entities whose targeting should be further explored. The activating or inhibiting, single or combinatorial, perturbations that prevent the activation of the proliferation and inflammatory markers and the inactivation of apoptotic and differentiation markers are presented in Table 1. The complete lists of perturbations are available in the accompanying Jupyter Notebook.

**Table 1:** Frequency of perturbations that prevent the system from reaching any of the dysregulated phenotypes in psoriasis

Perturbation A	Perturbation B	Frequency
VDR: 1	–	10
EP4: 0	–	9
EP3: 1	–	
PRKACA: 0	–	
NFKBIA: 1	–	8
NFKB: 0	–	
IKBKB: 0	–	
STAT3: 0	–	6
IFNGR: 0	–	5
IL17R: 0	–	4
cPLA2a: 0	–	
AA: 0	–	
PRKCA: 0	–	
PLCg: 0	PIK3R1: 0	
PLCg: 0	PDPK1: 0	

Single or combinatorial, activating or inhibiting perturbations as predicted by a perturbation analysis using Pint.

Generally, the analysis identified the targeting of key inflammatory regulators as the most impactful, especially those that are converging points of multiple pathways and receptors that appear to regulate critical entities for the behavior of the system. Vitamin D analogues such as calcipotriol have been widely used to treat moderate to severe psoriasis, with generally high effectiveness (Kim, 2010). It comes as no surprise, therefore, that the activation of VDR, the receptor activated by calcipotriol, stands out as a key node that can resolve the aberrant phenotypes represented in our model.

Other important perturbations included the inhibition of the EP4 receptor or the activation of the EP3 receptor confirming the important role of PGE_2_ and, subsequently, of cPLA_2_ɑ in the development of a psoriatic phenotype. As endogenous PGE_2_ is not directly activating the EP receptors in these perturbation simulations, but that their activation is fixed in the model when setting the input for the simulations, we suggest that when the tool predicts a perturbation that includes EP receptors, cPLA_2_ɑ inhibition could be expected to have the same, or at least similar, effect in the system. The inhibition of cPLA_2_ɑ or AA production indeed also appears among the beneficial results, although with a lower frequency. In addition to being proposed as a potential drug target by the model, EP4 is under investigation for the treatment of cancer, and its inhibition alone or combined with well-established treatments has shown promising results (Konya et al., 2013; Yamamoto et al., 2020). The EP3 receptor is a low-affinity PGE_2_ receptor, with distinct downstream signaling, as it acts through the inhibition of cAMP production. As a low-affinity receptor, EP3 is activated by low levels of PGE_2_ and, therefore, mainly associated with noninflammatory physiological states (Rundhaug et al., 2011). Although its exact role in skin physiology remains unclear, several studies have reported the involvement of EP3 in the inhibition of keratinocyte growth (Konger et al., 2005) and in restricting dendritic cell recruitment and functions *in vitro* (Shiraishi et al., 2013), both being attractive results in the context of psoriasis. Based on our model's predictions, and experimental observations, the direct or indirect disruption of PGE_2_ signaling in systems where inflammation is a driver could be a promising option for treatment and should be further tested. The next proposed perturbation was PRKACA (protein kinase cAMP-activated catalytic subunit alpha), with the same frequency as EP4. In the model, PRKACA exerts its effect mainly by regulating key survival pathways and regulators, such as the MAPKs and CREB1. Although its involvement in the regulation of proliferative pathways and psoriasis has been documented (Gudjonsson et al., 2010), the prediction that it could serve as an additional target that could reduce keratinocyte hyperproliferation in psoriasis is novel and not yet described in the literature.

NF-κB is a convergence point in the inflammatory response and directly regulates the transcription of many inflammatory genes. Therefore, the finding that its direct inhibition, or indirect inhibition through its regulators, can restore a more normal phenotype in KC, comes as no surprise. The importance of NF-κB as a key transcription factor in chronic inflammatory diseases, including psoriasis (Goldminz et al., 2013), has made NF-κB and its regulators an attractive target for treatment in many diseases (Gilmore and Herscovitch, 2006). Its inhibition in combination with the inhibition of other transcription factors such as STAT3 has also been explored (Andrés et al., 2013). Although the combination of NF-κB and STAT3 is not detected as an impactful perturbation, the inhibition of STAT3 singly has the next highest frequency in the list. The model proposes the inhibition of STAT3 as well as the inhibition of other entities that control many inflammatory genes that sustain the inflammatory cycle of psoriasis and have been previously studied as potential drug targets. These entities include IL-17 (Amin et al., 2018), which is targeted by several biological therapeutics used in psoriasis (Ly et al., 2019), and IFNγ. Although IFNγ exerts an important influence on the system, its inactivation alone is not enough to convert the psoriatic phenotypes back to normal. Its inhibition together with other entities such as IL-17, however, appears to be more effective (Meephansan et al., 2017). At the bottom of the list, some perturbations suggest the targeting of PI3K/AKT pathway components together with PLCγ. Both pathways are linked to proliferation and cell survival (Castilho et al., 2013; Haase et al., 1997).

## Discussion

In this study, we aimed to develop an executable logical model for investigating the regulation of keratinocyte physiology by pro-psoriatic cytokines that could be used to investigate the therapeutic mode of action of cPLA_2_α inhibitors in psoriasis and predict likely druggable combinatorial partners for future investigations. Our model, supported by primary experimental observations, suggests that cPLA_2_α-dependent PGE_2_/EP4 signaling is important for maintaining the psoriatic phenotype of KCs under cytokine stimulation, thus providing a therapeutic mode of action for the cPLA_2_α inhibitor AVX001 in psoriasis. Furthermore, we suggest AVX001 and the topical antipsoriatic drug calcipotriol have mainly distinct mechanisms of disease resolution; however, they commonly regulate the expression of some key signaling entities, and we predict beneficial therapeutic effects when used in combination.

The pathology of psoriasis involves an interaction between immune cells and epidermal KCs that is critical for maintaining the chronic disease state, with keratinocyte activation leading to the release of chemokines and cytokines that promote the infiltration and amplification of immune cells (Lowes et al., 2014). In our analysis we have focused on the key players that drive a vicious cycle of inflammation: the eicosanoids, produced by KCs and that act on KCs themselves and on immune cells, Th1-derived cytokines, which are established activators of phospholipases and cause eicosanoid release from KCs (Sjursen et al., 2000; Thommesen et al., 1998), and Th17-derived cytokines, which have largely unknown effects on eicosanoid signaling.

Our experimental findings demonstrated the importance of Th17-regulated PGE_2_ release via cPLA_2_ɑ in the induction of proinflammatory cytokine expression in KCs and suggest a role in cell survival. The dependence of IL-17 responses on PGE_2_ signaling was previously shown in normal human epidermal KCs and demonstrated to involve the activation of the MAPK pathway (Kanda et al., 2004, 2005). Because cPLA_2_ɑ is a well-known target of the MAPK pathway (reviewed in Clark et al. [1995]), this presents a putative mechanism for its regulation by Th17 cytokines. Th17 cytokines also increased *PTGER4*, which in turn can modulate cell survival and proliferation via PI3K (Peng et al., 2017) and ERK (Fujino et al., 2003) signaling. This suggests that Th17 cytokines may also regulate how the KCs respond to PGE_2_ and supports evidence that signaling via EP4 may predominate in psoriasis (Lee et al., 2019). Interestingly, calcipotriol suppressed the Th17-dependent release of PGE_2_, but not the increased abundance of *PTGS2*, and counteracted the changes in *PTGER4* and *PTGER2* expression. The suppressive effects of calcipotriol on PGE_2_ release differs from previous studies, showing that calcipotriol can stimulate or augment PGE_2_ release in both KCs and immune cells (Ashcroft et al., 2020; Ravid et al., 2016) and is rather consistent with the inhibition of Th17-dependent pro-inflammatory responses as described by Lovato et al. (2016). It will be interesting to investigate the mechanism of suppression of Th17-dependent PGE_2_ release by VDR activation, given it may be a novel mechanism accounting for some of the anti-psoriatic effects of the compound. The experimental data thus suggest both the cPLA_2_ɑ-COX2-PGE_2_ synthesis pathway and PGE_2_/EP4 signaling pathways are active in psoriatic KCs, with implications for both paracrine and autocrine signaling. The impact of autocrine EP4 signaling was further investigated using the psoKC model. Stable state analysis revealed that the states of key inflammatory and proliferation markers were active, corroborating their reported state in psoriasis only when EP4 was encoded to be active. We thus hypothesized that activation of the PGE_2_/EP4 axis is involved in an intrinsic amplification loop that could enable or sustain the effects of psoriatic cytokines and that suppression of the PGE_2_/EP4 axis by inhibition of cPLA_2_α presents a putative mode of action for AVX001 in psoriasis. We further explored this hypothesis using the *in vitro* model system by analyzing the expression of a subset of the phenotypic marker genes assigned as outputs and by exploring the behavior of the KCs in different conditions using our computation model.

Phenotypic characterization of the *in vitro* model showed its inability to recapitulate the hyperproliferative state of KCs in response to Th17 stimulation, which is a prominent feature of psoriatic skin. Evidence for the usefulness of such epidermis-equivalent cultures to recapitulate the hyperproliferative state of KCs in response to cytokine stimulation is lacking, indicating a potential requirement of either the dermal compartment or alternative immunological signaling molecules to recreate this aspect of the disease (Desmet et al., 2017). The computational model, on the other hand, accurately predicted the hyperproliferative state of the KCs in response to Th17 cytokines, allowing us to investigate the potential implication of inhibiting the cPLA_2_ɑ/PGE_2_/EP4 axis for a wider range of psoriatic phenotypes. Furthermore, the psoKC model was able to accurately reproduce the majority of our *in vitro* observations, in addition to those reported in the literature. We further explored its predictive use by expanding the simulations of the system's response to additional stimuli that were not tested in the lab, namely IFNγ and TNFα. The ability to capture a more representative cytokine microenvironment for psoriatic KCs, along with the integration of the cPLA_2_ɑ/PGE_2_/EP4 axis gave us access to an in silico experimentation system that allowed us to test a wide range of stimuli, observe the propagation of signals through the system, and predict the effects of mutations.

Logical models have been previously used to elucidate drugs' mechanisms of action and effectiveness (Béal et al., 2021; Traynard et al., 2017). In silico treatment with the drugs tested *in vitro* revealed distinct mechanisms of action and interactions between the cPLA_2_ɑ inhibition and vitamin D analogues, where the two drugs impacted different phenotypic aspects of KCs. Calcipotriol can have both an antiproliferative (Kristl et al., 2008; Liang et al., 2017) and a pro-apoptotic effect (Huang et al., 2019; Tiberio et al., 2009) in psoriatic KCs. The model predicted that calcipotriol, alone or in combination with cPLA_2_ɑ inhibition, acts either through rescuing the differentiation phenotype and/or via the induction of apoptosis. Alternatively, the inhibition of cPLA_2_ɑ signaling directly impacts the proliferative phenotype of the simulated psoriatic KCs, as already described by Ashcroft et al. (2020). In addition, as initially hinted by the *in vitro* experiments, the combination of the two treatments uncovered a common regulation of several system components related to both KC physiological state and inflammatory immune response.

We further demonstrated how a logical model can be used to better understand a system and its internal regulatory mechanisms. As proposed by Thieffry et al., the presence of negative circuits (i.e. negative feedback loops) in a model is expected to generate cyclic attractors (Thieffry, 2007), where the state of the nodes is oscillating (i.e. continuously alternating between active and inactive states). Remarkably, the model contained a very limited number of functional circuits. This attribute became apparent during the integration of psoriasis-specific information in the model. A characteristic example is the negative feedback loop between STATs and SOCSs, where STAT1 and STAT3 activate their inhibitors SOCS1 and SOCS3. However, the activation of SOCS1 and SOCS3 appears inadequate to completely inhibit the expression of STAT-downstream targets in psoriasis (Madonna et al., 2012), making this negative circuit nonfunctional in the context of psoriasis, and it was, therefore, removed from the model. This observation on the model's behavior proposes the dysregulation of mechanisms that would otherwise limit the spread of inflammatory response that might contribute to the development of psoriasis. Furthermore, stochastic simulations together with value propagation analyses confirmed the distinct role of Th1 and Th17 cells in the pathophysiology of psoriasis. All results indicated that Th1-derived cytokines have a key role in stimulating and further enhancing immune responses by regulating the majority of immune markers and chemotactic eicosanoids, as described in (Albanesi et al., 2018). The activation of cytokines and chemokines related to the recruitment, survival, and maintenance of Th17 and Th22 subpopulations by Th1 cells may indicate that Th1 activation precedes the activation of Th17 and Th22 during the development of psoriasis or that Th1 activation functions to amplify Th17 and neutrophil responses, as proposed in Kryczek et al. (2008). The value propagation results also corroborated the dominance of an IFNγ gene signature, where IFNγ dominated the regulation of markers and TNFα had a more limited impact. This observation supports the claims that TNFα is a potentiator and amplifier of IFNγ effects (Albanesi et al., 2018). The synergistic effect of TNFα with IL-17 has also been described (Chiricozzi et al., 2011). These integrative responses could explain why the influence of TNFα on other nodes is overlapping on regulations from other inputs. However, it is worth noting that TNFα plays an important role in the development of the disease and that its targeting remains a promising therapeutic option, but mainly due to its effect on Th17 cells (Furiati et al., 2019; Yost and Gudjonsson, 2009).

Predictive logical models have been previously used to propose treatment strategies that could affect the system in a desired way. The suggested perturbations included both known and novel targets, some of them already involved in well-established psoriasis treatments. For the rest of the targets, inhibitory agents are available, and sometimes even approved as drugs, possibly opening attractive opportunities for drug repurposing. The fact that many of the suggested perturbations concern already used or explored targets confirms that the model sufficiently integrates the role of those entities in the system and can recognize the importance of certain stimuli and pathways to the progression of the disease. For example, VDR activation by the use of vitamin D analogues and the blocking of IL-17 signaling are widely used in the treatment of psoriasis (Kim, 2010; Ly et al., 2019). The importance of the cPLA_2_ɑ/PGE_2_/EP4 axis was further highlighted by the proposal of components of the pathway as a way to restore a normal phenotype. The role of EP4 was previously speculated to be involved in psoriasis and KCs but is not fully explored for its potentials for treating the disease. Therefore, we further propose the exploration of the role of EP4 in psoriasis and its potential as a drug target. The activation of the less studied EP3 receptor appears to also be able to promote a closer-to-normal phenotype, and the role of agonists toward the receptor should be further studied, together with its general role in the skin (patho)-physiology. Another entity, the catalytic subunit of protein kinase A (PRKACA), was proposed as a promising drug target. PRKACA's downstream effects involve multiple pathways, including WNT signaling, a pathway directly related with cPLA_2_ɑ signaling (Xu et al., 2019), and psoriasis in general (Gudjonsson et al., 2010). PRKACA has also been studied in the context of cancer, where its aberrant regulation and overexpression have been related to inflammatory activation of Caspase 1, oncogene activation, and elevated PGE_2_ levels (Almeida et al., 2011). In addition to its role in hyperproliferation and inflammation, PRKACA has been associated with drug resistance in breast cancer by supporting the restoration of anti-apoptotic phenotypes of cancer cells (Moody et al., 2015). The current knowledge on the activity of the kinase, together with the model's predictions on the effects of its inhibition in psoriasis, suggest that its exact role in the development of the disease, together with its potential importance as a prognostic marker and/or drug target should be further explored.

Although the model identifies certain perturbations as potentially useful for therapeutic use, the need for further assessment of the results based on current knowledge and their actual experimental testing remains. For instance, the targeting of PLCγ together with components of the PI3K/AKT pathway is proposed by the model; this comes as no surprise, as their action affects proliferation and cell survival. However, PLCγ has been described as “undruggable” in the literature (Lattanzio et al., 2013), as the development of small molecule inhibitors against it appears troublesome. The blocking of IFNγ was also among the proposed perturbations. Even though its importance on the progression of the disease is supported by both the model and prior knowledge, targeting of IFNγ on its own fails to restore a normal phenotype (Meephansan et al., 2017). Some plausible explanations for the discrepancies between observed and predicted behaviors have been further discussed in the limitations of the study section of the paper.

### Conclusions

In conclusion, this paper demonstrates how a combination of *in vitro* and in silico models, both without doubt flawed in their accuracy of representing the system to be analyzed, can complement each other to test and generate hypotheses for characterizing the regulatory mechanisms and effects of stimuli in a cellular system. The use of the in silico model allowed us to interpret *in vitro* observations in a more holistic manner providing additional mechanistic details of drug actions and led us to propose novel candidates of drug targets that should be further explored. We characterized the regulation of certain lipid mediators in psoriatic KC, which, together with the prior knowledge of the involvement of cPLA_2_ɑ signaling, revealed yet another layer of involvement of KCs in the chronic inflammatory loop of psoriasis. In addition, we presented a computational framework to interpret the effect of chemical perturbations and various stimuli in a cellular network by exploring the differences between the mechanisms of actions of cPLA_2_ɑ inhibitors and calcipotriol. A perturbation analysis revealed promising entities whose targeting could restore a normal phenotype in KCs. For many of these targets, inhibitory agents have already been described, and sometimes these are even available as approved drugs, possibly opening attractive opportunities for drug repurposing. The combination of model-based analyses showcases how a systems biology approach can support a better understanding of a disease, further drive hypotheses on its causes, and suggest new targets for potential treatment.

### Limitations of the study

Although the logical model of psoriatic keratinocytes appears to have a high predictive value, there are certain limitations to it, mainly related to the discrete nature of the modeling, its dependence on prior knowledge, and its focus specifically on keratinocytes.

First, the discretization method of the experimental results together with the intrinsic abstraction of discrete models could lead to some loss of information about the state of an entity. For instance, we observed experimentally that calcipotriol treatment reduced the expression of Th17-stimulated *IL1B* expression but the effect was not significant and therefore did not meet the threshold set during the discretization process for inhibition. At the same time, the model predicts that IL-1β will be completely inhibited by calcipotriol. This discrepancy could be explained by the inability of the logical model to distinguish between partial and full inhibition or that the threshold for discretization based on the experimental observations was too stringent. In addition, as a gatekeeper of inflammation, IL-1β is under a strict regulation involving post-translational cleavage of its precursor, proIL-1β, for complete activation (Liu et al., 2016). The control of calcipotriol over this mechanism of activation has been described in other systems, such as hematopoietic stem cells (Wang et al., 2020) and is thus included in the PKN but not captured by measuring gene expression experimentally. Although there was an extensive effort to incorporate the regulatory interactions relevant to eicosanoid signaling reported in the literature, half of the discrepancies involve the state of markers upon treatment with cPLA_2_ɑ inhibitors. Such predictions may result from the focus of the model on signaling mediated by PGE_2_ and not by other eicosanoids or from gaps in the current knowledge on how cPLA_2_ɑ-derived lipid signaling mediators influence KCs. Lastly, despite the model providing some insights on intracellular communication by integrating the effects of immune-cell-derived cytokines as stimuli and KC-derived secreted factors that influence immune cells, it likely covers only a fraction of the complex intra- and inter-cellular signaling that underlies psoriasis. It is, therefore, expected that some observations at the tissue level cannot be captured to their totality by single-cell models. Although cell-specific models, as the one presented, aids the understanding of the role and behavior of individual cells in a disease, multicellular models are likely to provide a more accurate representation of biology, as they would capture emergent behaviors that arise from intercellular interactions and communications.

## STAR★Methods

### Key resources table

**Table:** 

REAGENT or RESOURCE	SOURCE	IDENTIFIER
**Antibodies**		

Cytokeratin 10 Monoclonal mouse antibody (DE-K10)	Invitrogen	CAT # MA5-13705 RRID:AB_90109722
sMonoclonal mouse anti-human Ki-67 Antigen- Clone MIB-1	Dako	CAT # M7240 RRID:AB_86403
EnVision GΙ2 Double stain system Rabbit/mouse (DAB+/Permanant Red)	Agilent- Dako	CAT # K5361

**Chemicals, peptides, and recombinant proteins**		

Interleukin 17A	Cell Signalling Technologies	CAT # 8928SC
Interleukin 22	Cell Signalling Technologies	CAT # 8931SC
human recombinant epidermal growth factor	Sigma Aldrich	CAT# E9644
AVX001 Storage at -80 °C as a 20 mM stock solution in DMSO under argon gas to minimize oxidation	Dr. Inger Reidun Aukrust and Dr. Marcel Sandberg, Synthetica AS, Norway	CAS # 300553-18-8
Calcipotriol hydrate	Sigma-Aldrich	CAS # 112965-21-6

**Critical commercial assays**		

Enzyme-linked immunosorbent assay for LTB_4_	Cayman	CAT # 10009292
Enzyme-linked immunosorbent assay for 12s-HETE	Enzo Lifesciences	CAT # ADI-900-050
Enzyme-linked immunosorbent assay for prostaglandin E2	Cayman	CAT # 514435

**Deposited data**		

Logical model of psoriatic keratinocytes - psoKC model	This paper.	https://www.ebi.ac.uk/biomodels/MODEL2109300001 and https://doi.org/10.5281/zenodo.5549353

**Experimental models: Cell lines**		

HaCaT	Prof. N. Fusenig (Heidelberg, Deutsches Krebsforschungszentrum, Germany)	RRID: CVCL_0038

**Oligonucleotides**		

Primers	See Table S1 for primer sequences	

**Software and algorithms**		

bioLQM	http://colomoto.org/biolqm/	https://doi.org/10.3389/fphys.2018.01605
GINsim	http://www.colomoto.org/software/ginsim.html	https://doi.org/10.1016/j.biosystems.2009.04.008
MaBoSS	http://www.colomoto.org/software/maboss.html	https://doi.org/10.1186/1752-0509-6-116
Pint	http://www.colomoto.org/software/pint.html and https://loicpauleve.name/pint/	
Value propagation algorithm	Hernandez et al., 2020	https://doi.org/10.3389/fphys.2020.558606
PsoKC model analysis code	This paper	https://doi.org/10.5281/zenodo.5549353 and https://github.com/Eirinits/psoKC_model
qBASE+ version 3.2	https://www.qbaseplus.com/	
LinRegPCR version 2017.1	https://medischebiologie.nl/files/	https://doi.org/10.1093/nar/gkp045
GraphPad Prism version 7	https://www.graphpad.com/	

### Resource availability

#### Lead contact

Further information and requests for resources and reagents should be directed to and will be fulfilled by the lead contact, Eirini Tsirvouli (eirini.tsirvouli@ntnu.no.

#### Materials availability

This study did not generate new unique reagents

### Experimental model and subject details

#### HaCaT keratinocytes

The spontaneously immortalized skin KC cell line HaCaT is derived from the epidermis of a caucasian male (Boukamp et al., 1988) and was kindly provided by Prof. N. Fusenig (Heidelberg, Deutsches Krebsforschungszentrum, Germany). HaCaT are a commonly used cell system to study proliferative and inflammatory responses in psoriasis research (Farkas et al., 2003; Feuerherm et al., 2013; Lehmann, 1997; Pozzi et al., 2004; Se et al., 2010; Ziv et al., 2008); the cells differentiate on reaching confluency and form stratified epithelia when grown under appropriate conditions (Hinitt et al., 2011; Schoop et al., 1999). The HaCaT cells used in this study were not authenticated.

HaCaT cells were maintained in Dulbecco's modified Eagle Medium (DMEM) supplemented with 5% (v/v) FBS, 0.3 mg/mL glutamine, and 0.1 mg/mL gentamicin (DMEM-5) at 37°C with 5% CO2 in a humidified atmosphere. Cultures were maintained at sub-confluency (below 95%) by splitting (1:5) every 3-4 days. 3D culture of HaCat KCs was carried out using the 24-well carrier plate system from Thermo Fisher Scientific (#141002) according to the protocol described in (Ashcroft et al., 2020). The culture inserts (0.4 μm pore size) were coated using the Coating Matrix kit (Thermofisher Scientific #R-011-K) according to the manufacturer's protocol. HaCaT were seeded at a density of 0.3 × 10^5^ cells per insert in 0.5 mL DMEM-5 and incubated for 24 h before being lifted to the air-liquid interface. The media in the lower chamber was replaced with DMEM-5 (without antibiotics) + 1 ng/mL EGF and 5 μg/mL L-ascorbic acid in the absence or presence of Th17 psoriatic cytokines (IL-17, 10 ng/ml and IL-22, 10 ng/ml) and either AVX001 (5 μM), calcipotriol (10 nM) or a combination of AVX001 (5 μM) and calcipotriol (10 nM). The media in the lower chambers was changed every 3rd day for 12 days.

### Method details

#### RNA extraction and quantitative PCR

Total RNA was extracted from HaCaT 3D cultures using the RNeasy kit (QIAGEN) according to the manufacturer′s protocol. The amount and purity of the RNA samples were quantified using a Nanodrop One/OneC Microvolume UV–VIS Spectrophotometer (ND-ONE-W) from ThermoFisher Scientific. RNA samples with absorbance (A) 260/280 between 2.0 and 2.2 were accepted. 1 μg of total RNA per sample was reverse transcribed using the QuantiTect Reverse Transcription Kit (Qiagen, Hilden, Germany) according to the manufacturer's protocol. Quantitative PCR was performed with the LightCycler 480 SYBR Green I Master MIX and LightCycler 96 instrument from Roche (Basel, Switzerland) according to the manufacturer's protocol using primer concentrations of 0.5 μM each. The amplification protocol started with an initial step of 95°C (10 min) followed by 45 cycles of denaturation at 95°C (10 s), annealing at 55°C (10 s) and elongation at 72°C (10 s). Melting analysis was performed using the following parameters: 95°C for 5 s, 65°C for 1 min, 97°C for 1 min and cooling at 50°C for 10 s. Three reference genes were selected for gene expression analysis: TBP, HPRT1, and GAPDH as recommended for keratinocytes (Allen et al., 2008). All primers were KiCqStart primers designed by and purchased from Sigma Aldrich and the sequences are listed in Table S1.

#### Immunohistochemistry

We determined the proportion of proliferating cells and cells undergoing differentiation in the 3D HaCaT cultures using ki67 and cytokeratin 10 (CK10) immunostaining respectively. The 3D cultures were fixed by immersion in 4% paraformaldehyde (PFA) overnight; the membranes were removed from the inserts and prepared in Tissue Clear (Sakura, Osaka, Japan) for paraffin wax embedding using the Excelsior AS Tissue processor (ThermoFisher Scientific). Paraffin-embedded sections (4 μm) were cut onto SuperFrost Plus slides (ThermoFisher scientific), dried overnight at 37°C, and then baked for 60 min at 60°C. The sections were dewaxed in Tissue Clear and rehydrated through graded alcohols to water in an automatic slide stainer (Tissue-Tek Prisma, Sakura). Next, the sections were pretreated in Target Retrieval Solution, High pH (Dako, Glostrup, Denmark, K8004) in PT Link (Dako) for 20 min at 97°C to facilitate antigen retrieval. The staining was performed according to the manufacturer's procedure with EnVision G|2 Doublestain System Rabbit/Mouse (DAB+/Permanent Red) kit (Dako/Agilent K5361) on the Dako Autostainer. Endogenous peroxidase and alkaline phosphatase activity were quenched with Dual Endogenous Enzyme Block (Dako). Sections were then rinsed in wash buffer and incubated with primary antibody against Ki67 (MIB1: Dako M7240), diluted 1:300, for 40 min. The slides were rinsed before incubating in horseradish peroxidase (HRP) - polymer and 3,3′-Diaminobenzidine (DAB) to develop the stain. After a double stain block, the sections were incubated in antibody against cytokeratin 10 (Invitrogen #MA5-13705), diluted 1:100, for 60 min. After incubation in the mouse/rabbit linker, the sections were incubated in AP- polymer and the corresponding red substrate buffer with washing between each step. Tris-buffered saline (TBS; Dako K8007) was used throughout the washing steps. The slides were lightly counterstained with hematoxylin, completely dried, and coverslipped. Appropriate negative controls were performed; both mouse monoclonal isotype control (Biolegend, San Diego, CA) and omitting the primary antibody (negative method control).

#### Enzyme-linked immunosorbent assay

For measurement of eicosanoid levels, supernatants from the lower chambers of the HaCaT 3D cultures were removed at day 12, just prior to fixation or harvesting of the cells, and analyzed by enzyme-linked immunosorbent assay (ELISA) for PGE_2_, LTB_4_, and 12s-HETE according to the manufacturer's protocols (see key resources table for details). Undiluted samples were hybridized overnight, and the enzymatic conversion of the substrate was read at OD420 nm.

#### Prior knowledge network of psoriatic KCs

A Prior Knowledge Network (PKN) encompassing the main signaling events taking place in KCs during the chronic stage of psoriasis was manually curated based on prior knowledge of the disease. The PKN consists of biological entities (*nodes*) with a reported role in the disease and their regulatory interactions (*edges*), as identified by an extensive and careful assessment of the available literature and knowledge extraction from databases with causal molecular interactions, such as SIGNOR (Perfetto et al., 2016). The curation was focused on the signaling cascades initiated by the main cytokines that characterize chronic psoriatic lesions, namely IL-17, IL-22, TNFα, and IFNγ. Additionally, as the model aimed to explore the role of cPLA_2_a and eicosanoids in psoriatic KC signaling, the metabolism and signaling interactions involving eicosanoids with a reported role in psoriasis and those identified through our *in vitro* experiments were added to the model to allow for hypothesis-driven simulations. Lastly, signaling through the Vitamin-D receptor (VDR) was included to allow the exploration of the potential combinatorial effect between cPLA_2_ɑ inhibition and vitamin D analogues like calcipotriol. Each signaling component was annotated by its official gene symbol, UniProt ID or ChEBI ID, while all interactions were annotated with the PubMed IDs supporting their addition to the model.

#### The psoriatic keratinocyte logical model

Following the construction of the PNK, the network was converted to a Boolean model and encoded in the GINsim software (Naldi, 2018). In Boolean models, the activity of a node (also called *state*) can be associated with Boolean values (i.e. 1 or 0). Nodes with a value of 1 are considered active while a value of 0 is associated with inactive nodes. Lastly, for each node, the logical rules governing its state were specified. The logical rules aimed to capture how a node's state depends on the state and combinations of its regulators and were manually specified following the logical formalism AND, OR, and NOT. The definition of the rules was an iterative process based on the results of various model analyses and their comparison to the expected behavior described in the literature and our *in vitro* observations.

#### Model analysis

The final model was analyzed using software tools developed by the CoLoMoTo consortium (http://www.colomoto.org/). The analyses explored the system from several angles aiming i) to assess its performance and validate its closeness to biological reality, ii) to use the model as a tool to understand how cPLA_2_ɑ downstream lipid mediators and more specifically PGE_2_, together with Th17 and Th1 cytokines contribute to the different phenotypes of the disease, and iii) to explore how the targeting of cPLA_2_ɑ alone or together in treatments with vitamin D analogues affects the system. The model analysis in full is performed and archived as a Jupyter Notebook (Tsirvouli and Kuiper, 2021).

#### Model validation and stable state prediction

To validate the performance of the model, with respect to its fidelity to biological reality and ability to correctly predict experimental observations, the stable states or attractors (comparable to cellular states, or phenotypes) of the model under various conditions were computed using the bioLQM toolkit (Naldi, 2018). The model contains a subset of nodes that can be used as markers of specific physiological states (e.g. proliferation, survival, apoptosis or differentiation). The state of these marker-nodes was used to infer the cellular states that the model can emulate under various conditions (e.g. external stimuli and perturbations). The ratio between the activating and inhibiting states of markers depends on the model's response to specific inputs and dictates which phenotypes will be reached. For instance, whether a cell will undergo apoptosis does not depend on the activation of pro-apoptotic regulators, but rather on the ratio between pro-apoptotic (e.g. Bax) and anti-apoptotic proteins (e.g. BCL-2) (Khodapasand et al., 2015)

The model's validation and analyses were performed in three main conditions, which are summarized below. In addition to the conditions tested *in vitro* (i.e. the Th17-derived IL-17 and IL-22), a simulation more representative for the psoriatic milieu, where IL-17, IL-22, TNFα, and IFNγ are present, was also included. Additionally, to elucidate the potential distinct role of the Th17- and Th1-derived cytokines, the behavior of the model in the presence of the Th1 (i.e., IFNγ and TNFα) and Th17 cytokines separately was explored. Lastly, as EP4 appears to be regulated in response to IL-17 and IL-22 (experimental observations presented in the “*Integration of experimental observations into the model and validation with in vitro results*” section), the receptor was encoded to be active in the analyses.

The three psoriatic conditions that were chosen for the stable state calculations are summarized below:

• TH17: Only Th17 cytokines are present, and EP4 is activated (i.e. IL17R, IL22R and EP4 input nodes are ON).
• TH1: Only Th1 cytokines are present, and EP4 is activated. (i.e. TNFRSF1A, IFNGR and EP4 input nodes are ON).
• ALL: All Th17 and Th1 cytokines are present, and EP4 is activated (i.e. IL17R, IL22R, TNFRSF1A, IFNGR and EP4 input nodes are ON).

After the validation of the model against experimental observations, the behavior of the system under different chemical perturbations was simulated. In all of the three above conditions (i.e. TH17, TH1, ALL), three perturbations were performed:

• **AVX**: Inhibition of cPLA2 (i.e. cPLA2 node set to 0)
• **CAL**: Activation of VDR receptor (i.e. VDR node set to 1)
• **COMBO**: Combination of AVX and CAL (i.e. cPLA2 node set to 0 and VDR node set to 1)

Regulatory circuits and their functionality were analyzed in order to investigate the existence of multiple attractors and/or cyclic attractors. Functional positive circuits (or *positive feedback loops*) can give rise to multiple attractors, while functional negative circuits (or *negative feedback loops*) can result in cyclic attractors (Thieffry, 2007).

#### Stochastic simulations

The probabilistic interpretation of node states, phenotypes, and their potential transient behavior were explored with stochastic simulations using MaBoSS (Stoll et al., 2017). Additionally, the ‘time’-dependent evolution of the markers' states together with the probability of reaching a phenotype were analyzed. MaBoSS allows the calculation of phenotype probabilities when model variables, such as node states or inputs, are altered, allowing the comparison of how the behavior of a system depends on those changes. Stochastic simulations were performed for three conditions; with only Th17 cytokines, with only Th1 cytokines, and with all cytokines present. The analysis was performed to identify whether the evolution of the system could vary depending on the inputs, or whether the system would reach a psoriatic state with either of the two sets of cytokines. To ensure that the whole state space was explored, a high number of trajectories (10.000) was chosen. The fraction of instances (from a total of 10.000) a state is reached is taken as the probability of that state. In order to explore the activation profiles of each node in response to the different stimuli, the initial state of the nodes was set as inactive, while only certain input nodes, chosen based on condition to be simulated, were active. However, in a cell population cells may occupy various states with different sets of entities active in each cell, which may result in a different response to the same input, depending on the specific state of that cell. For that reason, the dependency of the results on the nodes' initial states was also explored.

#### Value propagation analysis

A comparison of the impact of Th17- and Th1-derived cytokines was investigated by a value propagation study, as described in (Hernandez et al., 2020). The same analysis was also performed for each cytokine individually. The latter comparisons were conducted to identify whether cytokines produced by the same Th-cell populations have complementary or overlapping actions and to compare the influence of each cytokine on its own. In value propagation analysis, a value of 1 is assigned to a node of interest (emulating its activity), in our case the different cytokine receptors of the model, and propagated through the model. This allows the identification of those nodes that are bound to be active or inactive in one of the two compared conditions or in both, and, therefore, aids the comparison of the impact of specific inputs to the system. While setting a value of 1 to the node(s) of interest, the value of the VDR node was set to 0 in all comparisons. This ensured that the analysis would include the influence only of the disease-related nodes, as the effect of Vitamin D analogues had been encoded in the model as an input node that regulates the state of downstream nodes as any other.

#### Perturbations restoring a normal phenotype

Finally, a perturbation analysis was performed in order to explore the complete node perturbation space (both singly and combinatorial) and identify potential perturbations (activating or inhibiting) that would drive the diseased system in a preferred direction and drug targets that could potentially be explored as treatment options. This was performed as a mutation analysis with the Pint tool (Paulevé, 2017), which identifies perturbations that will prevent the activation of the proliferation and inflammatory markers, and the inactivation of apoptotic and differentiation markers, essentially signifying the return to a normal KC phenotype. Pint provided a list of combinations for each of the markers. The combinations were then analyzed and ranked for the frequency that a single or combinatorial perturbation was proposed. It should be noted that the tool might propose combinations that are in fact redundant, meaning that the addition of a second perturbation will have the same impact as the individual one. The presented list in the results is curated to include only those perturbations with a distinct impact on the system.

### Quantification and statistical analysis

#### Immunohistochemistry

Brightfield images taken at 400× magnification using the EVOS FL Auto imaging system were used to determine the thickness, and the proliferation and differentiation of the 3D cultures. Manual measurement of thickness was performed in ImageJ (http://imagej.nih.gov/ij). The total number of cells (based on hematoxylin counterstain of the nuclei), Ki-67 positive, and CK-10 positive cells were counted per image and used to determine the Ki67 index (Ki-67 positive count/total count) and CK10 index (CK10 positive count/total count.) The data are typically based on >20 images per replicate and are reported as the mean ± SEM of three biological replicates per treatment group. One-way ANOVA followed by Dunnett's multiple comparison test was used to make statistical comparisons. Graphing and statistical analysis was performed in GraphPad Prism version 7.

#### Enzyme-linked immunosorbent assay

ELISA data were processed using a 4-parameter logistic fit model using MyAssays (myassays.com) to provide concentrations for PGE_2_, LTB_4_ and 12S-HETE in pg/ml. The average of two samples per biological replicate was taken and the data are reported as the mean ± SEM for three biological replicates. One-way ANOVA followed by Dunnett's multiple comparison test was used to make statistical comparisons of the levels of PGE_2_ between treatment groups. For LTB_4_ and 12sHETE, some values were below the detection limit of the assay. Where values were below the detection limit, we substituted a value equal to half the detection limit of the assay in further analyses, and the Kruskal-Wallis testfollowed by Dunn's multiple comparison test was used to make comparisons. All statistical analyses were performed in GraphPad Prism version 7.

#### Quantification of relative gene expression

Ct values and PCR amplification efficiencies were calculated from the raw amplification curves using LinRegPCR software (version 2017.1) (Ruijter et al., 2014; Tuomi et al., 2010) and subsequently imported into qBASE+ version 3.2 for quantification of relative expression and statistical analysis (Hellemans et al., 2007). Expression has been normalized to three reference genes (TBP, HPRT1 and GAPDH) and the data are presented as the mean calibrated normalized relative quantity (CNRQ) scaled to the untreated control (CTRL) ± SEM for three biological replicates. ANOVA, corrected for multiple testing using the Tukey-Kramer method, was used to generate two-sided p values. For data discretization, p < 0.05 was considered to represent a significant difference between control and treatment groups. Statistical analysis was done in qBASE+ version 3.2.

#### Experimental data discretization

In order to make the expression level measured by qPCR comparable to the discrete (i.e. 0 and 1) states that the model predicts, a discretization step was performed. As there is no specific threshold for inferring the state of the different gene products, the state was based on the relative expression of the genes. The discretization workflow is presented in Figure S3 in the supplemental information.

## Data Availability

• Relative gene expression data have been deposited at Mendeley and are publicly available as of the date of publication. Immunohistochemistry images and other experimental data are available from the lead author upon request. • All original code for the analysis of the model has been deposited at Zenodo and is publicly available as of the date of publication. DOIs are listed in the key resources table. • Any additional information required to reanalyze the data reported in this paper is available from the lead contact upon request.

## Supplemental information

Document S1. Figures S1–S8 and Tables S1 and S3

Table S2. Model node annotation and logical rules, related to Figure 2

## Footnotes

Supplemental information can be found online at https://doi.org/10.1016/j.isci.2021.103451.
