# Supplementary material for: Logical and experimental modeling of cytokine and eicosanoid signaling in psoriatic keratinocytes
Source: iScience. 2021 Nov 15;24(12):103451. doi: 10.1016/j.isci.2021.103451 (PMC8633970; doi:10.1016/j.isci.2021.103451)
Supplement: Document S1. Figures S1–S8 and Tables S1 and S3 [file mmc1.pdf]

iScience, Volume *24*

Supplemental information

Logical and experimental modeling of cytokine and eicosanoid signaling in psoriatic keratinocytes

Eirini Tsirvouli, Felicity Ashcroft, Berit Johansen, and Martin Kuiper

Figure S1. [Quantitative PCR of PGE2-pathway components], Related to Figure 1.

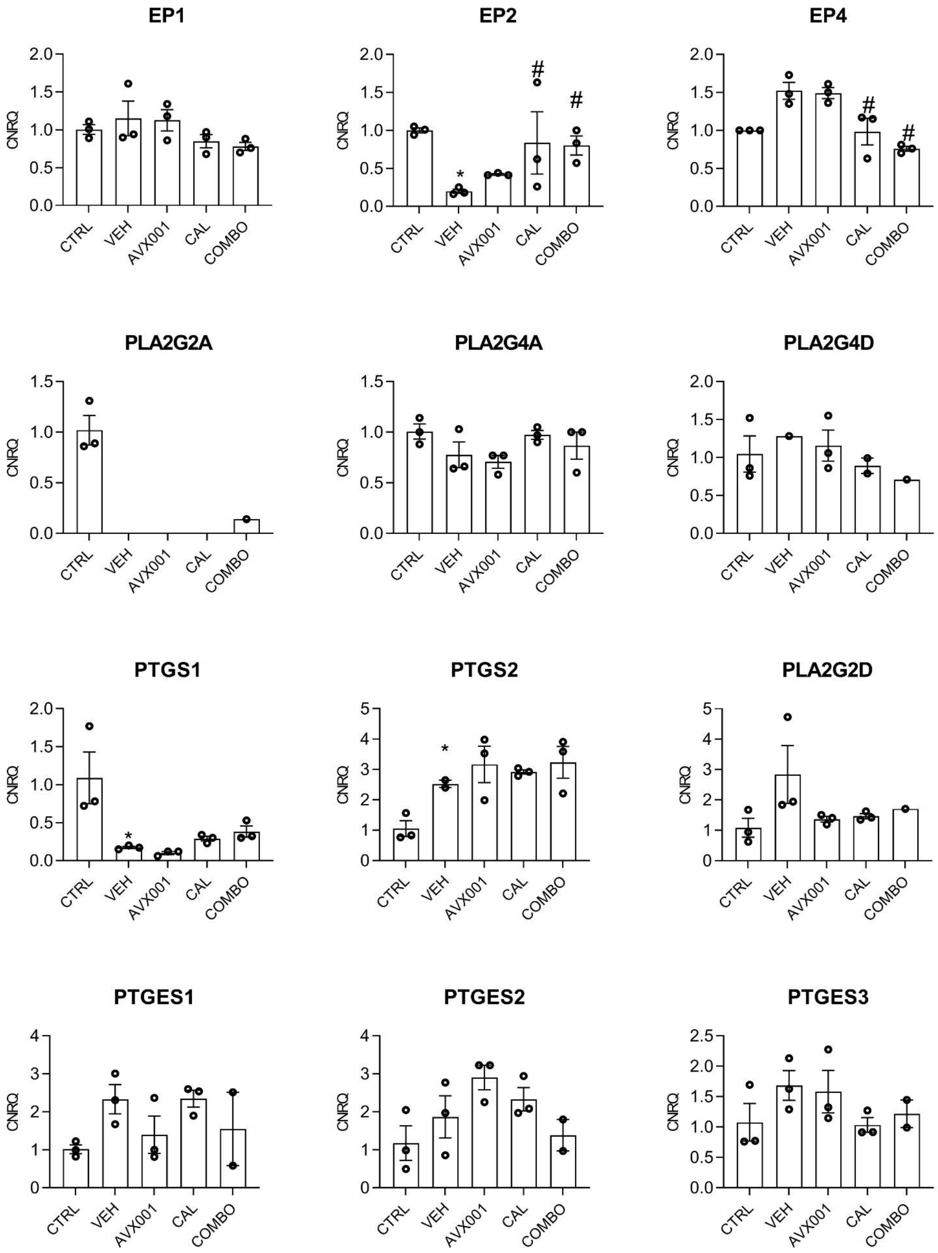

Figure S1. Quantitative PCR analysis of selected PGE2 synthesis, degradation and signaling pathway components. Data shown are the comparative normalized gene expression levels (CNRQ) relative to unstimulated controls (CTRL) for cultures treated with Th17 cytokines alone (VEH)or in the presence of AVX001 (5 µM), calcipotriol (10 nM) CAL, or a combination of AVX001 and calcipotriol (COMBO). Individual data point are shown, and the bars are the mean ∓ SEM. Statistical significance was calculated by one-way ANOVA with Dunnett's post analysis; *p<0.05 versus unstimulated control (CTRL) # p<0.05 versus vehicle treated control (VEH).

Figure S2. [Quantitative PCR of model output nodes], Related to Figure 3.

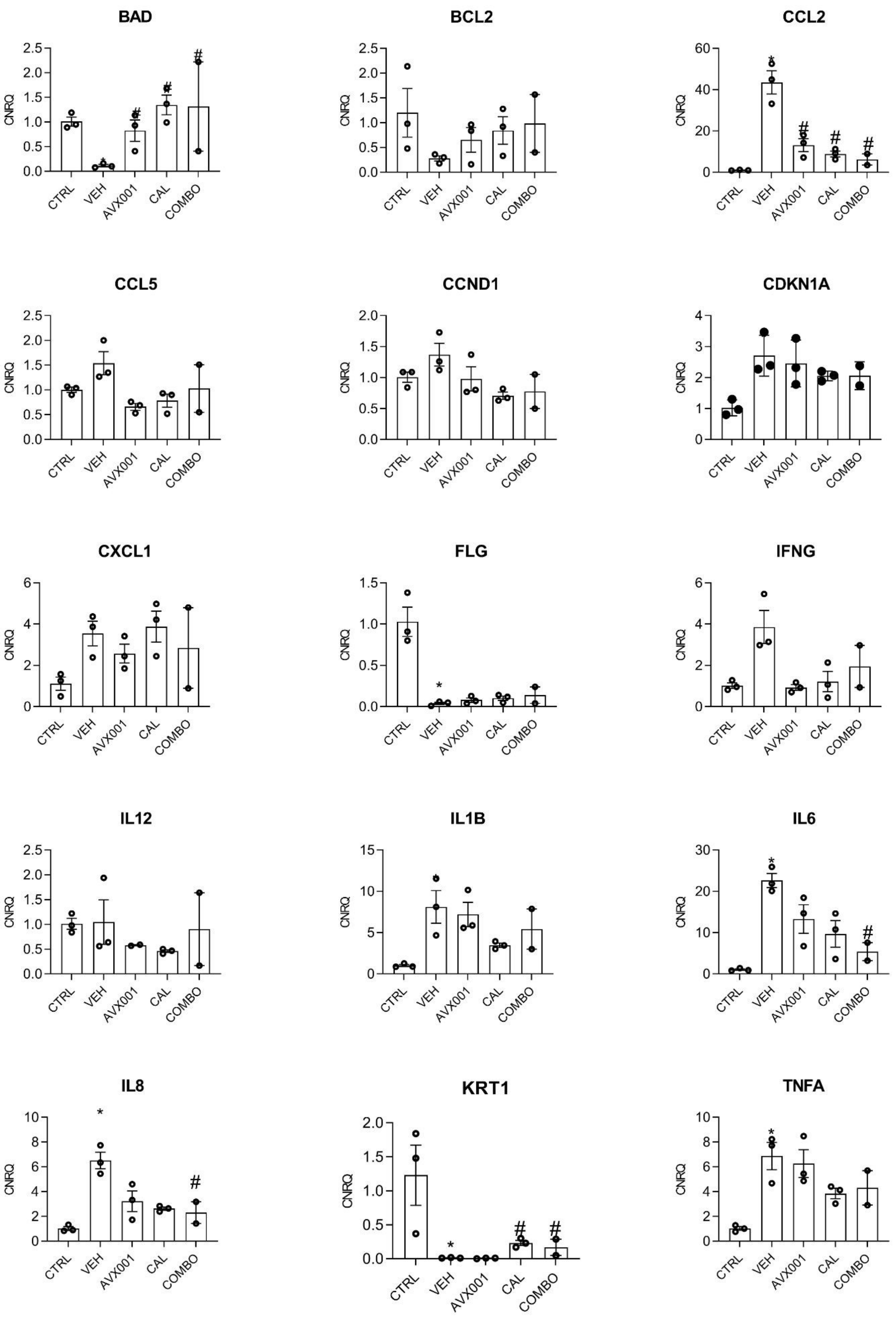

Figure S2. Quantitative PCR analysis of selected model output genes. Data shown are the comparative normalized gene expression levels (CNRQ) relative to unstimulated controls (CTRL) for cultures treated with Th17 cytokines alone (VEH) or in the presence of AVX001 (5 µM), calcipotriol (10 nM) CAL, or a combination of AVX001 and calcipotriol (COMBO). Individual data point are shown, and the bars are the mean ∓ SEM.Statistical significance was calculated by one-way ANOVA with Dunnett's post analysis; *p<0.05 versus unstimulated control (CTRL) # p<0.05 versus vehicle treated control (VEH).

Figure S3. [Experimental data discretization], Related to STAR Methods and Figure 3.

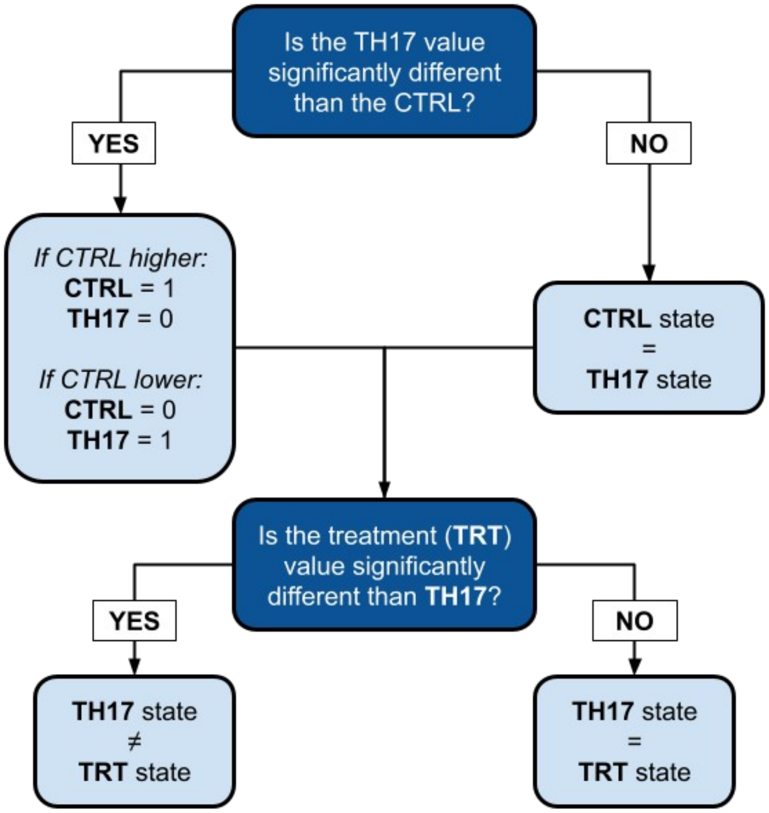

Figure S3: Schematic workflow of discretization process of experimental results. **TH17:** Expression value after treatment with IL-17 and IL-22. **TRT:** Expression value after treatment with AVX001, Calcipotriol, or their combination.

Figure S4. [Phenotype probability after the stimulation with IFNg and TNFa], Related to "*Evolution of the regulatory system through "time", and phenotype probabilities*" result section.

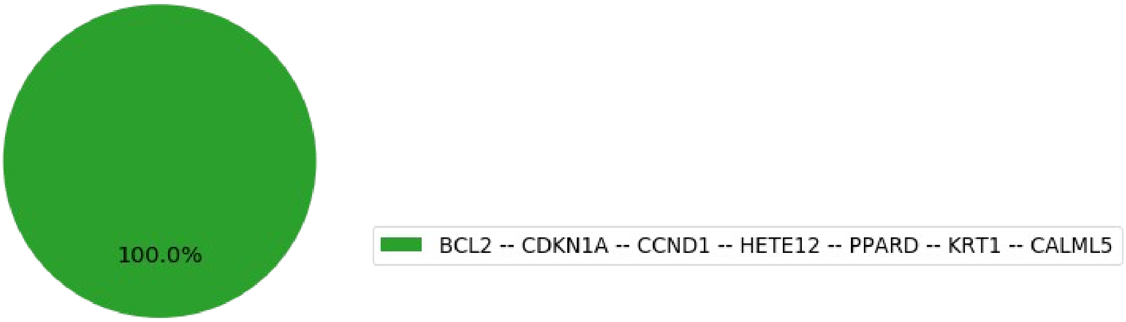

Figure S4. Phenotype probability pie chart. Probabilities of stable states after the stimulation with IFNg and TNFa in synchronized cell populations as calculated by stochastic simulations. Only selected survival and apoptosis markers are shown.

Figure S5. [State trajectory of cell fate markers], Related to "*Evolution of the regulatory system through "time", and phenotype probabilities*" result section.

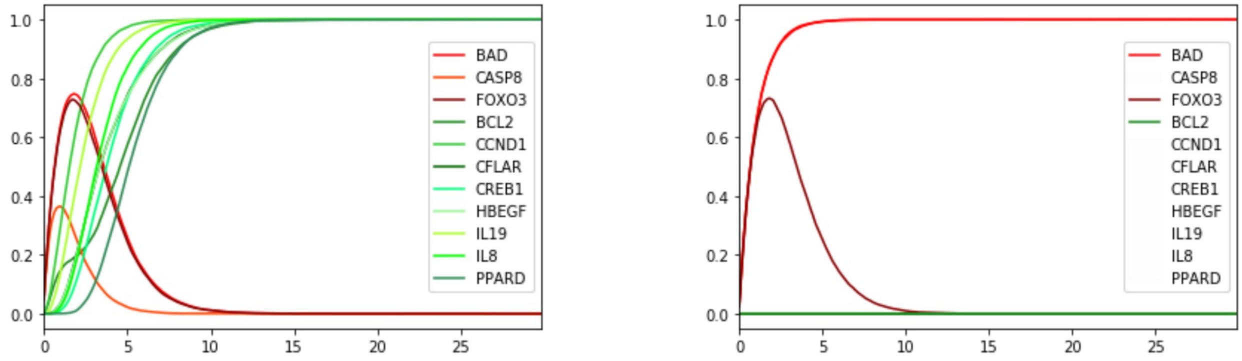

Figure S5. Plots of the state trajectory of cell fate markers in i) untreated IL-17, IL-22, TNFα and IFNγ stimulated cells (left panel) and ii) treated with cPLA2 inhibitor and vitamin D analogs IL-17, IL-22, TNFα and IFNγ stimulated cells (right panel). Green hue lines = proliferation and survival markers, Red hue lines = Apoptosis markers. Nodes with a common trajectory after treatment (right panel) are presented with a single line in a graph.

Figure S6. [Value propagation graphs for Th1- and Th17-derived cytokines], Related to Table 1

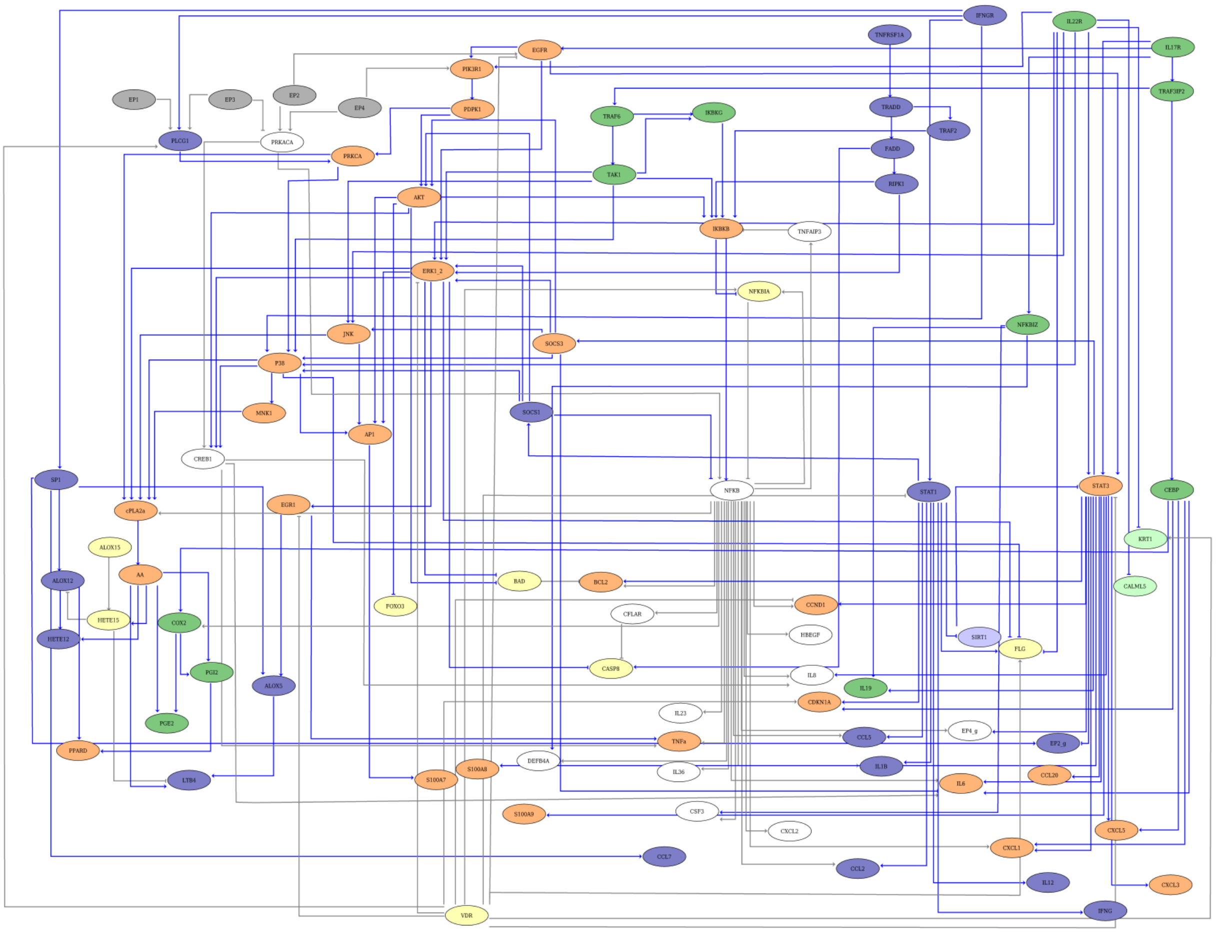

Figure S6. Comparative figure of value propagation results between Th1- and Th17-derived cytokines. The colors represent a node's activation in one of both conditions. **White** = Free in both, **Light yellow** = OFF in both, **Light orange** = ON in both, **Light purple** = OFF within TH1 cytokines, **Dark purple** = ON within TH1 cytokines, **Light green** = OFF within Th17 cytokines , **Dark green** = ON within Th17 cytokines.

Figure S7. [Value propagation graphs for IL-17 and IL-22], Related to Table 1

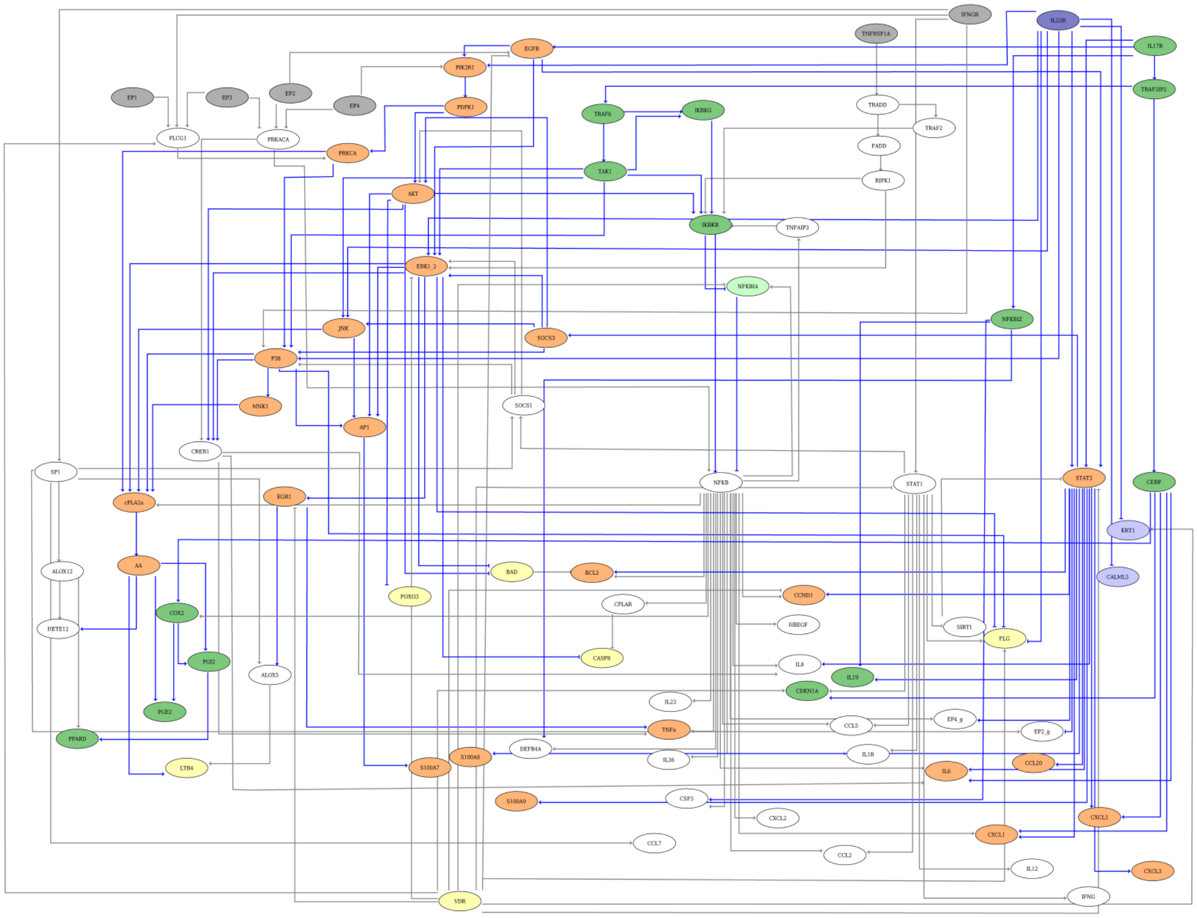

Figure S7. Comparative figure of value propagation results between IL-17 and IL-22. The colors represent a node's activation in one of both conditions. **White** = Free in both, **Light yellow** = OFF in both, **Light orange** = ON in both, **Light purple** = OFF within IL-22 cytokines, **Dark purple** = ON within IL-22 cytokines, **Light green** = OFF within IL-17 cytokines , **Dark green** = ON within IL-17 cytokines.

Figure S8. [Value propagation graphs for IFNg and TNFa], Related to Table 1

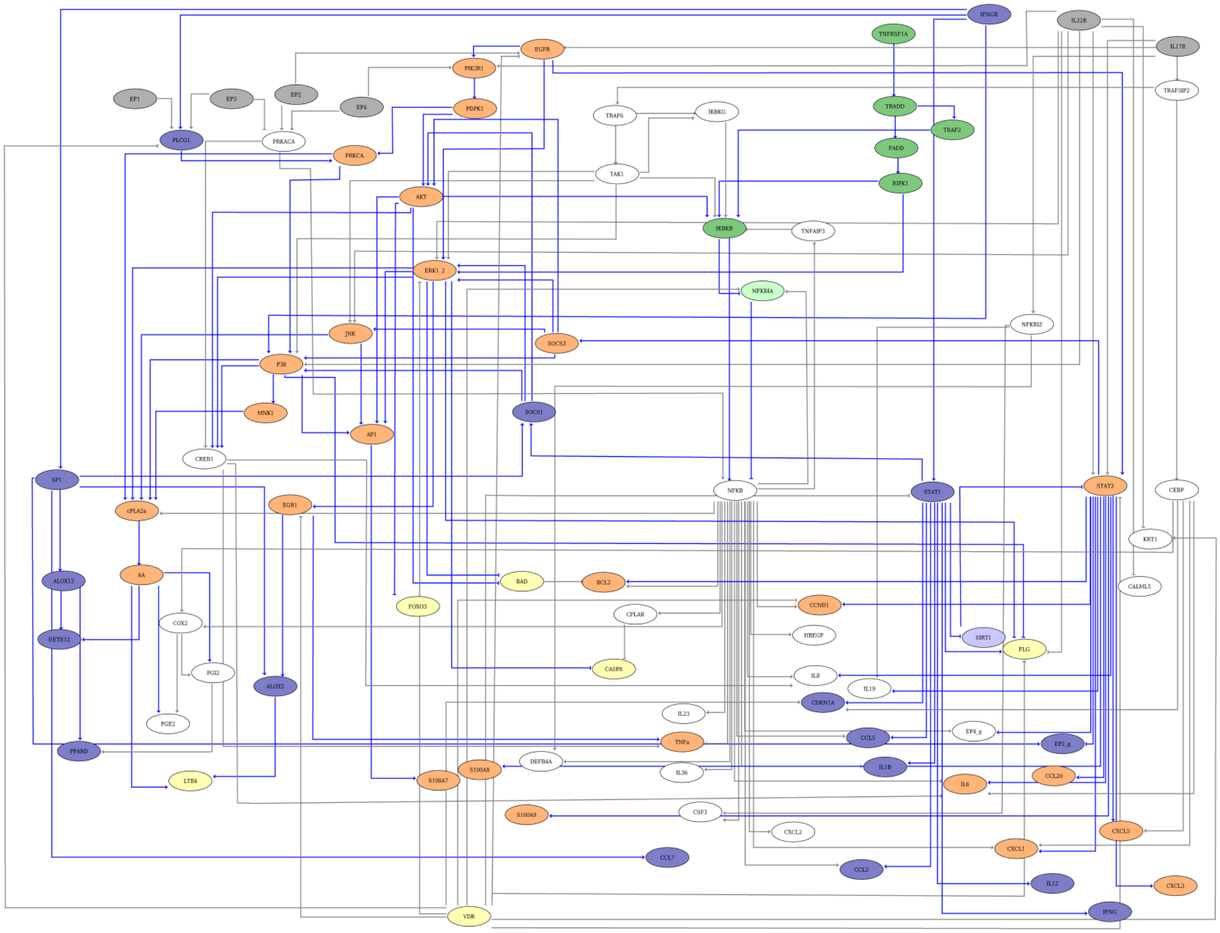

Figure S8. Comparative figure of value propagation results between IFNg and TNFa. The colors represent a node's activation in one of both conditions. **White** = Free in both, **Light yellow** = OFF in both, **Light orange** = ON in both, **Light purple** = OFF within IFNg cytokines, **Dark purple** = ON within IFNg cytokines, **Light green** = OFF within TNFa cytokines , **Dark green** = ON within TNFa cytokines.

Table S1. [Primer sequences], Related to STAR Methods

Gene	Primer sequences	
GAPDH	*Forward*	5'-ACAGTTGCCATGTAGACC-3'
	Reverse	5'-TTTTTGGTTGAGCACAGG-3'
CCL2	*Forward*	5'-AGACTAACCCAGAAACATCC-3'
	Reverse	5'-ATTGATTGCATCTGGCTG-3'
PLA2G2A	*Forward*	5'-GGAAAAAGAGCAACAGATCC-3'
	Reverse	5'-GAGGACTCCAGAGTTGTATC-3'
PLA2G4A	*Forward*	5'-CTTGATACTCCAGATCCCTATG-3'
	Reverse	5'-GAAATGTCTTGTTCTCTTCCTG-3'
PTGS1	*Forward*	5'-GTTCTGGGAGTTTGTCAATG-3'
	Reverse	5'-GAGTGTAATAGCTCACGTTG-3'
PTGS2	*Forward*	5'-AAGCAGGCTAATACTGATAGG-3'
	Reverse	5'-TGTTGAAAAGTAGTTCTGGG-3'
CCND1	*Forward*	5'- GCCTCTAAGATGAAGGAGAC-3'
	Reverse	5'-CCATTTGCAGCAGCTC-3'
CDK1	*Forward*	5'-ACCTATGGAGTTGTGTATAAGG-3'
	Reverse	5'-GACTGACTATATTTGGATGACG-3'
CCL5	*Forward*	5'-AAGTCTCTAGGTTCTGAGC-3'
	Reverse	5'-TTTTATGGTTGCATTGAGAAC-3'
IL6	*Forward*	5'-GCAGAAAAAGGCAAAGAATC-3'
	Reverse	5'-CTACATTTGCCGAAGAGC-3'
IL8	*Forward*	5'-GTTTTTGAAGAGGGCTGAG-3'
	Reverse	5'-TTTGCTTGAAGTTTCACTGG-3'
KRT1	*Forward*	5'-GAGGATATAGCCCAGAAGAG-3'
	Reverse	5'-ATCTAAGTCTCTGGATCACAC-3'

HPRT1	*Forward*	5’-ATAAGCCAGACTTTGTTGG-3’
	Reverse	5’-ATAGGACTCCAGATGTTTCC-3’
TBP	*Forward*	5’-GCCAAGAGTGAAGAACAG-3’
	Reverse	5’-GAAGTCCAAGAACTTAGCTG-3’
PTGER1	*Forward*	5'- CACTTCTAAGCACAACCAG-3'
	Reverse	5'- CGCAGAATGGCTTTTTATTC-3'
PTGER2	*Forward*	5'- CTTGCCTTTCACGATTTTTG-3'
	Reverse	5'- AAAAACCTAAGAGCTTGGAG-3'
PTGER3	*Forward*	5'- TTTTGCCAGGAGGAATTTTG-3'
	Reverse	5'- CAAAAAGAGAGTCATGGAG-3'
PTGER4	*Forward*	5'- AAGGGCTATCATCATCCTAC-3'
	Reverse	5'-TCTAGTAGAAAAACGGAGG -3'
PLA2G4D	*Forward*	5’-CGTCAGATCGCCCAGAAAAC-3’
	Reverse	5’-CCAAGGAAGGATGTCTGTGTGT-3’
PTGES1	*Forward*	5’-CAAAAACATCACTCCCTCTC-3’
	Reverse	5’-AAAAGTCTGCATTCTTAGCC-3’
PTGES2	*Forward*	5’-CTGTACTGACTCTAGGAAGG-3’
	Reverse	5’-TTTAGTTGTTGCGAGCTTTC-3’
PTGES3	*Forward*	5’-CAGATGATGATTCACAAGA-3’
	Reverse	5’-CTTTAGAGCTATCAACTCA-3’
PGDH	*Forward*	5’-TTGGTTTCTGTTATCAGTGG-3’
	Reverse	5’-ATATTGATAATGATGCCGCC-3’
BAD	*Forward*	5’-ATCATGGAGGCGCTG-3’
	Reverse	5’-CTTAAAGGAGTCCACAAAC-3’
BCL2	*Forward*	5’-GATGGGAACACTGGTGGAGGATGG-3’

	Reverse	5’-TCTGGAGGGCCCACGGCAG-3’
CXCL1	*Forward*	5’-ATGCTGAACAGTGACAAATC-3’
	Reverse	5’-TCTTCTGTTCCTATAAGGGC-3’
FLG	*Forward*	5’-AATTTCGGCAAATCCTGAAG-3’
	Reverse	5’-CTTGAGCCAACTTGAATACC-3’
IFNG	*Forward*	5’-GGTAACTGACTTGAATGTCC-3’
	Reverse	5’-TTTCGCTTCCCTGTTTTAG-3’
TNFA	*Forward*	5’-TACTCCCAGGTCCTCTTCAAG-3’
	Reverse	5’-GATGCGGCTGATGGTGTG-3’
IL1B	*Forward*	5’-CTAAACAGATGAAGTGCTCC-3’
	Reverse	5’-GGTCATTCTCCTGGAAGG-3’
IL12a	*Forward*	5’-AAGACCTCTTTTATGATGGC-3’
	Reverse	5'- CATTCATGGTCTTGAACTCC-3'

Table S3. [Marker-nodes which were used to define the model's physiological state and their associated phenotypes and processes], Related to STAR Methods, Figure 2 and Figure 3.

Nodes in red-colored cells are inhibiting the phenotype associated with their respective column. Nodes with an asterisk (*) were experimentally tested for the validation of the model's performance.

Keratinocyte markers			Inflammatory & immunostimulatory markers				
Proliferation & Survival	Differentiation	Apoptosis	Inflammation	Th1	Th17	Other immune cells	PGE2-related
**CCND1***	**FLG***	**BAD***	S100A7	**IFNG***	**IL-1B***	**IL6***	**PTGER2_g***
**CXCL8/ IL8***	**KRT1***	CASP8	S100A8	CXCL3	**TNFA***	**CXCL1***	**PTGER4_g***
**PGE2***	**CDKN1A***	**BCL2***	S100A9	IL12	**CCL2***	CCL7	
IL19 **	SIRT1	CFLAR	DEFB4A		**CCL5***	CXCL2	
12-HETE	CALML5				CCL20	CXCL5	
PPARD	IL36				IL23	LTB4	
HBEGF	TRAF3IP2				IL36	CSF3	
CDKN1A							
SIRT1							

** IL-19 activates fibroblasts to produce keratinocyte growth factor, which promotes keratinocyte proliferation.
